# Biocatalytic Syntheses of Antiplatelet Metabolites of the Thienopyridines Clopidogrel and Prasugrel Using Fungal Peroxygenases

**DOI:** 10.3390/jof7090752

**Published:** 2021-09-13

**Authors:** Jan Kiebist, Kai-Uwe Schmidtke, Marina Schramm, Rosalie König, Stephan Quint, Johannes Kohlmann, Ralf Zuhse, René Ullrich, Martin Hofrichter, Katrin Scheibner

**Affiliations:** 1 Institute of Biotechnology, Brandenburg University of Technology Cottbus-Senftenberg, Universitätsplatz 1, 01968 Senftenberg, Germany; kai-uwe.schmidtke@b-tu.de (K.-U.S.); marina.schramm@b-tu.de (M.S.); rosalie.koenig@b-tu.de (R.K.); katrin.scheibner@b-tu.de (K.S.); 2 Fraunhofer Institute for Cell Therapy and Immunology, Branch Bioanalytics and Bioprocesses, Am Mühlenberg 13, 14476 Potsdam-Golm, Germany; 3 Chiracon GmbH, Im Biotechnologiepark 9, 14943 Luckenwalde, Germany; quint@chiracon.de (S.Q.); kohlmann@chiracon.de (J.K.); zuhse@chiracon.de (R.Z.); 4 Department of Bio- and Environmental Sciences, TU Dresden-International Institute Zittau, Markt 23, 02763 Zittau, Germany; rene.ullrich@tu-dresden.de (R.U.); martin.hofrichter@tu-dresden.de (M.H.)

**Keywords:** thienopyridine, clopidogrel, prasugrel, unspecific peroxygenase, human drug metabolites, antiplatelet

## Abstract

Antithrombotic thienopyridines, such as clopidogrel and prasugrel, are prodrugs that undergo a metabolic two-step bioactivation for their pharmacological efficacy. In the first step, a thiolactone is formed, which is then converted by cytochrome P450-dependent oxidation via sulfenic acids to the active thiol metabolites. These metabolites are the active compounds that inhibit the platelet P2Y_12_ receptor and thereby prevent atherothrombotic events. Thus far, described biocatalytic and chemical synthesis approaches to obtain active thienopyridine metabolites are rather complex and suffer from low yields. In the present study, several unspecific peroxygenases (UPOs, EC 1.11.2.1) known to efficiently mimic P450 reactions in vitro—but requiring only hydroperoxide as oxidant—were tested for biocatalytic one-pot syntheses. In the course of the reaction optimization, various parameters such as pH and reductant, as well as organic solvent and amount were varied. The best results for the conversion of 1 mM thienopyridine were achieved using 2 U mL^−1^ of a UPO from agaric fungus *Marasmius rotula* (*Mro*UPO) in a phosphate-buffered system (pH 7) containing 5 mM ascorbate, 2 mM h^−1^ H_2_O_2_ and 20% acetone. The preparation of the active metabolite of clopidogrel was successful via a two-step oxidation with an overall yield of 25%. In the case of prasugrel, a cascade of porcine liver esterase (PLE) and *Mro*UPO was applied, resulting in a yield of 44%. The two metabolites were isolated with high purity, and their structures were confirmed by MS and MS^2^ spectrometry as well as NMR spectroscopy. The findings broaden the scope of UPO applications again and demonstrate that they can be effectively used for the selective synthesis of metabolites and late-state diversification of organic molecules, circumventing complex multistage chemical syntheses and providing sufficient material for structural elucidation, reference material, or cellular assays.

## 1. Introduction

**Scheme 1:**
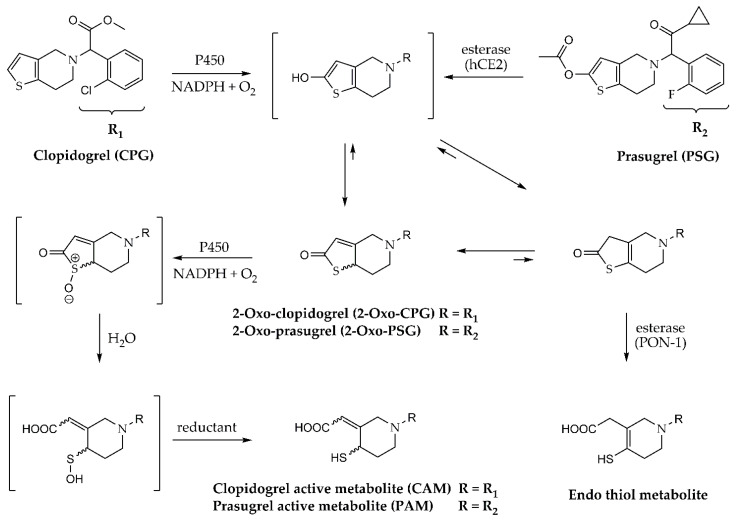
Proposed mechanism of bioactivation and conversion of clopidogrel and prasugrel into their active thiol metabolites, CAM and PAM, by cytochrome P450 enzymes via formation of thiolactone, thiolactone sulfoxides and sulfenic acids as intermediates. An alternative inactivation pathway has been described to proceed via hydrolysis catalyzed by thioesterases (PON-1) leading to the formation of the endo-thiol metabolite.

Clopidogrel (Plavix^®^, Iscover^®^) and prasugrel (Efient^®^) are antithrombotic prodrugs of the thienopyridine family that ignite their intrinsic effect after metabolic bioactivation and irreversibly inhibit the platelet P2Y_12_ receptor resulting in prevention of atherothrombotic events [1,2,3]. The two-step enzymatic bioactivation required for both prodrugs differs in its initial reactions: In the case of clopidogrel, the hydroxylation of the thiophene ring, required for the spontaneous formation of the thiolactone metabolite, 2-oxo-clopidogrel (Scheme 1), is catalyzed by cytochrome P450 (CYP) monooxygenases [4,5]. In case of prasugrel, the corresponsive thiolactone (R-95913) is formed by hydrolysis of its ester functionality, which seems to be mainly catalyzed by human carboxylesterases hCE2 [6]. The final transformation of these thiolactone derivatives into the respective active thiol metabolites is formally a thioester hydrolysis, since the oxidation state remains unchanged. In fact, thioesterases such as paraoxonase-1 (PON-1) are thought to accomplish this kind of hydrolysis, but the reactions studied led to the biologically inactive endo-isomer, in which the double bond has migrated into the piperidine ring [7,8]. Recent studies disclosed the formation of the active metabolite as a multistep biochemical process that also involves CYP enzymes, while the exact chemical nature of the intermediates is still under discussion. The most plausible metabolic process describes the formation of thiolactone sulfoxides during the oxidative activation, the hydrolytic opening of which leads to sulfenic acids that are efficiently reduced by ascorbate, phosphines, reductases or glutathione to give the active thiol metabolites (Scheme 1) [9,10,11,12].

The bioactivation of clopidogrel to form 2-oxo-clopidogrel and the active thiol metabolite can be catalyzed by several CYP enzymes. Kinetic and inhibition studies, however, revealed that just CYP2C19 contributes substantially to both oxidative steps and that CYP3A4 is essential for the formation of the active metabolite. Furthermore, CYP1A2, CYP2B6 and CYP2C9 may be involved in the oxidative cascade [5]. Several studies revealed that polymorphism in the gene encoding CYP2C19 contributes to the variability of platelet response to clopidogrel in patients. Individuals who carry a reduced-function *CYP2C19* allele had significantly lower levels of the active metabolite of clopidogrel, diminished platelet inhibition and a higher rate of major adverse cardiovascular events [13,14,15]. In addition, clopidogrel undergoes an extensive first-pass metabolism in the human liver where most of the prodrug is hydrolyzed to inactive clopidogrel carboxylic acid by carboxylesterase hCE1, followed by glucuronidation and renal excretion [16]. To address these causes for the low plasma level of active metabolites, prasugrel was developed replacing the ester functionality with a metabolically stable ketone and the introduction of an ester group in the thiophene 2-position that switched the first-step activation from CYP2C19 to carboxylesterase hCE2 [17].

**Figure 1:**
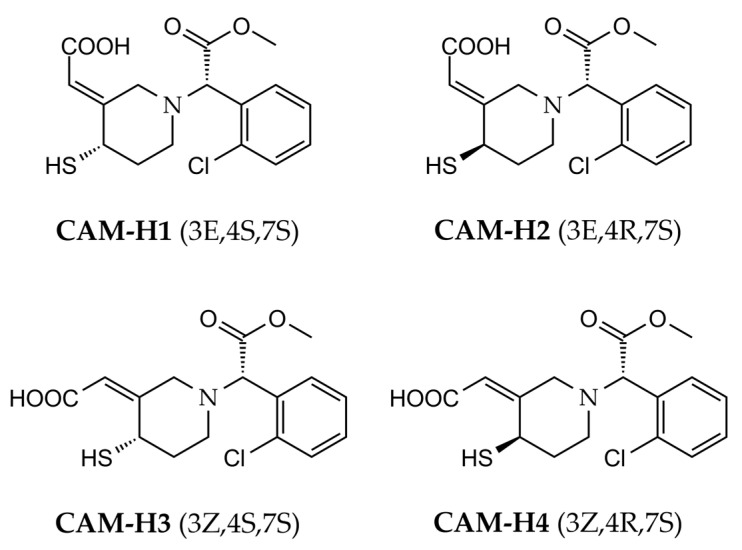
Structures of clopidogrel active metabolites H1–H4.

In vitro metabolism studies showed that metabolic activation of clopidogrel results in a mixture of four stereoisomers (H1–H4, Figure 1). The H1 and H2 isomers are *trans* isomers, whereas the exocyclic double bond between C3 and C16 in H3 and H4 appears in *cis* configuration. Analysis of clinical samples demonstrated that only H3 and H4 are formed in vivo, with only H4 being active. Therefore, the configuration of the thiol group at C4 seems to be crucial for antiplatelet activity [18]. The reactive free thiol group of the active metabolites leads to high instability in plasma samples, which is why they were stabilized by derivatization with 2-bromo-3’-methoxyacetophenone to be subsequently quantified by HPLC-MS [19]. For the unambiguous structural elucidation of metabolites H1–H4, syntheses for both the stabilized and free thiol metabolites have been described in the literature with yields below 3% [20,21]. Synthesis of active metabolites using human liver microsomes is hampered by low yield as well and by complicated purification protocols, and hence is not suitable for use on a preparative scale [18]. The active metabolite of prasugrel (PAM, R-138727) found in human plasma samples consists of four stereoisomers, (*R*, *S*)-, (*R*, *R*)-, (*S*, *S*)- and (*S*, *R*)-isomers (the first letter stands for the configuration of the thiol group and the second for that at the benzylic position), with 84% found as (*R*, *S*)- and (*R*, *R*)-, representing the most potent isomers. This underlines the importance of the *R*-configuration of the thiol group for bioactivity [22,23,24]. A chemical synthesis of PAM (R-138727) has not been reported so far. 

Herein, we report biocatalytic syntheses of the bioactive metabolites of clopidogrel and prasugrel by unspecific peroxygenases (UPOs, EC 1.11.2.1) secreted by fungi. UPOs have arisen to ‘dream catalysts’ for oxyfunctionalization reactions, since they incorporate selectively oxygen into nonactivated hydrocarbons generating hydroxylations and epoxidations that are difficult to attain by chemical methods [25,26,27]. These extracellular glycosylated heme-thiolate proteins are activated by hydrogen peroxide and work independently of electron donors, transport proteins and additional cofactors. Their catalytic cycle combines characteristics of heme peroxidases and cytochrome P450s, which is why UPOs and P450 enzymes have partly overlapping reaction portfolios [28,29]. UPOs have already been used for the conversion of various pharmaceuticals, e.g., volixibat (*N*-dealkylation), cyclophosphamide (aliphatic hydroxylation), propranolol (aromatic hydroxylation), testosterone (epoxidation) and corticosteroids (C–C fission) [30,31,32,33,34]. In this context, it was found that, depending on the catalyzed reactions, the most relevant parameters in respect to reaction optimization were pH, H_2_O_2_ concentration and dosing rate as well as organic solvents and their content. The effective generation of authentic samples of drug metabolites is important for their structural confirmation and for LC-MS recovery, for evaluation of their potential safety risk, for investigation of drug–drug interactions, for pharmacological testing and for detailed pharmacokinetic and pharmacodynamic analysis [26,30,31].

We demonstrate here the selective oxidation of thienopyridines and the subsequent ring opening of intermediary formed thiolactones into the bioactive 4-mercapto-3-piperidinylidene acetic acid derivatives, CAM and PAM, by UPOs.

## 2. Materials and Methods

### 2.1. Chemicals

Clopidogrel (methyl (2S)-2-(2-chlorophenyl)-2-(6,7-dihydro-4*H*-thieno[3,2-c]pyridine-5-yl) acetate; bisulfate) and prasugrel (5-(2-cyclopropyl-1-(3-fluorophenyl)-2-oxoethyl)-6,7-dihydro-4*H*-thieno[3,2-c]pyridine-2-yl acetate) were purchased from Acros Organics (Fair Lawn, NJ, USA) and 2-oxo-clopidogrel (methyl 2-(2-chlorophenyl)-2-(2-oxo-4,6,7,7α-tetrahydrothieno[3,2-c]pyridine-5-yl) acetate) was ordered from Santa Cruz Biotechnology (Dallas, TX, USA). All other chemicals were obtained from Merck Chemicals (Darmstadt, Germany) if not indicated otherwise. All chemicals had reagent-grade purity or were analytical standards.

### 2.2. Enzymes

The conversions of thienopyridines were carried out with wild-type UPOs (main isoforms) isolated from the cultures of *Agrocybe aegerita* DSM 22459 (*Aae*UPO, 46 kDa), *Coprinellus radians* DSM 888 (*Cra*UPO, 45 kDa), *Marasmius rotula* DSM 25031 (*Mro*UPO, 32 kDa), *Chaetomium globosum* DSM 62110 (*Cgl*UPO, 36 kDa) and *Marasmius wettsteinii* DSM 106021 (*Mwe*UPO, 32 kDa), which are deposited at the German Collection of Microorganisms and Cell Cultures (Braunschweig, Germany). The enzymes were produced and isolated as described previously [33,34,35,36,37]. Depending on the protein, UPOs were purified by fast protein liquid chromatography (FPLC) using a defined combination of size exclusion chromatography (SEC), ion exchange chromatography (IEC) and/or hydrophobic interaction chromatography (HIC). Enzyme purity was verified by sodium dodecyl sulfate polyacrylamide gel electrophoresis (SDS-PAGE) and UV/Vis spectroscopy. The specific activities of *Aae*UPO, *Cra*UPO, *Mro*UPO, *Cgl*UPO and *Mwe*UPO were 58, 26, 12, 7 and 16 U mg^−1^, respectively, whereas one unit (1 U) corresponds to the oxidation of 1 μmol of veratryl alcohol (3,4-dimethoxybenzyl alcohol) to veratraldehyde (3,4-dimethoxybenzaldehyde) in 1 min [1]. The formation of veratryl aldehyde was measured photometrically at 310 nm (*ε*_310_ = 9.3 mM^−1^ cm^−1^) in 50 mM potassium phosphate (KP_i_) buffer at pH 7.0 (*Aae*UPO, *Cra*UPO, *Cgl*UPO) or 5.5 (*Mro*UPO and *Mwe*UPO).

Porcine liver esterase (PLE) was purchased from Merck Chemicals (Darmstadt, Germany) with a specific activity of 18 U mg^−1^, where one unit represents the hydrolysis of 1 µmol ethyl butyrate in 1 min at pH 8.

All enzyme samples were diluted or dissolved in 10 mM KP_i_ buffer (pH 7 or 5.5) to obtain a volume activity of 20 U mL^−1^ before usage in small-scale reactions (500 µL).

### 2.3. Enzymatic Reactions

Typical UPO reactions (500 µL final volume) contained 1 mM of the respective thienopyridine, 10% acetone, 20 mM KP_i_ buffer (pH 7 or 5.5), 5 mM ascorbate and 2 U mL^−1^ UPO. The reaction mixture was tempered to 25 °C and the reaction kept running by adding aqueous H_2_O_2_ (0.25 µmol) in pulses every 15 min over a period of 2 h to a final concentration of 4 mM. The reaction mixtures with prasugrel (PSG) were preincubated with 2 U mL^−1^ PLE for 30 min before ascorbate, UPO and H_2_O_2_ were added. The influence of the organic cosolvent was investigated with acetone in the concentration range 0–40% (*v*/*v*) and by substitution of 10% acetone with acetonitrile (MeCN), methanol (MeOH), dimethyl sulfoxide (DMSO) or dimethyl formamide (DMF). As a reductant, glutathione (5 mM) was tested as an alternative to ascorbate, as well as the absence of such. All reaction setups were shaken at 700 rpm on a thermo shaker (TurboShaker 3500, Scienova GmbH, Jena, Germany) and stopped by adding 500 µL of ice-cold acetonitrile (−20 °C). Subsequently, the samples were centrifuged at 14,000× *g* for 10 min and the supernatants were analyzed by HPLC and MS. All reaction mixtures were performed in duplicate. Control experiments for every setup were carried out without UPO or without H_2_O_2_.

### 2.4. Semi-Preparative Synthesis of Thienopyridine Metabolites

#### 2.4.1. Clopidogrel Active Metabolite (CAM)

Clopidogrel bisulfate (CPG, 84 mg, 0.2 mmol) was dissolved in 40 mL acetone, 140 mL deionized water and 20 mL KP_i_ buffer (200 mM, pH 7) in a round-bottom flask. To this suspension, 350 mg ascorbate and 600 U *Mro*UPO (50 mg) were added, and the mixture was stirred at 25 °C and 200 rpm on a magnetic stirrer. Using a syringe pump, a 400 mM hydrogen peroxide solution was continuously added at a rate of 1 mL h^−1^ (equivalent to 2 mM h^−1^). Every 15 min, a 200 µL sample was taken, mixed with 200 µL of acetonitrile (−20 °C), centrifuged and analyzed by HPLC to monitor the reaction progress. Another 200 µL sample was taken to measure residual enzyme activity. The reaction was stopped after two hours by extracting the aqueous mixture with dichloromethane, and the organic layer was dried on anhydrous sodium sulfate and filtered. The organic solvent was removed under reduced pressure, the crude product was recovered as brownish oil (approx. 45 mg) and purified by preparative liquid chromatography. The clopidogrel active metabolite (CAM) was isolated as a mixture of isomers (molar ratio 42:58) in 13% yield (9 mg, 0.025 mmol) with a purity above 95% (HPLC). HR-ESI-MS: 356.0712 [M + H]^+^; theor. for C_16_H_18_ClNO_4_S + H^+^: 356.0723. ^1^H NMR (500 MHz, CDCl_3_) *δ* 7.55 (d, *J* = 6.9 Hz, 1H), 7.38 (d, *J* = 7.1 Hz, 1H), 7.29–7.22 (m, 2H), 5.56 (s, 0.6H), 5.45 (s, 0.4H), 5.23 (m, 1H), 4.79 (s, 1H), 3.69 (s, 3H), 3.60 (d, *J* = 12.9 Hz, 0.6H), 3.52 (d, *J* = 12.6 Hz, 0.4H), 3.18 (d, *J* = 13.0 Hz, 0.6H), 2.98 (d, *J* = 13.0, 0.4H), 2.91–2.71 (m, 0.8H), 2.65 (m, 1.2H), 2.26–2.12 (m, 1H), 2.10 (m, 1H), 1.78 (m, 1H). ^13^C NMR (126 MHz, CDCl_3_) *δ* 170.92, 170.49, 159.13, 134.71, 132.90, 129.98, 129.74, 129.59, 127.19, 114.39, 67.82, 53.67, 53.35, 52.23, 45.59, 45.24, 32.77, 31.71.

#### 2.4.2. 2-Oxo-Prasugrel (2-Oxo-PSG, R-95913)

Prasugrel (PSG, 150 mg, 0.4 mmol) was dissolved in 40 mL acetone, 140 mL deionized water and 20 mL KP_i_ buffer (200 mM, pH 7). Then, 22 mg PLE was added, and the suspension was stirred at 25 °C and 200 rpm. Thin-layer chromatography (TLC, ethyl acetate/n-hexane 1:1) indicated a complete conversion after 60 min. The reaction mixture was extracted with dichloromethane, and the organic phase was dried with sodium sulfate. Evaporation in vacuo gave 135 mg of a yellow pale oil that was further purified by preparative liquid chromatography; yield: 110 mg of isomers (molar ratio 53:47) of 2-oxo-PSG (83%, >98% purity). HR-ESI-MS: 332.1115 [M + H]^+^; theor. for C_18_H_18_FNO_2_S + H^+^: 332.1121. ^1^H NMR (500 MHz, CDCl_3_) *δ* 7.37–7.23 (m, 2H), 7.20–7.05 (m, 2H), 5.99 (s, 1H), 4.82 (s, 1H), 4.06 (m, 1H), 3.90 (m, 1H), 3.11–3.00 (m, 1.5H), 2.81 (d, 0.5H), 2.49 (td, 0.5H), 2.38–2.23 (m, 1.5H), 2.06 (m, 1H), 1.95–1.79 (m, 1H), 1.06–0.93 (m, 2H), 0.91–0.73 (m, 2H). ^13^C NMR (126 MHz, CDCl_3_) *δ* 207.2, 198.78, 167.86, 162.33, 160.38, 130.88, 130.51, 126.67, 124.62, 116.11, 120.84, 71.08, 53.30, 51.40, 51.07, 50.71, 48.66, 34.07, 18.78, 12.25, 11.77.

#### 2.4.3. Prasugrel Active Metabolite (PAM, R-138727)

Prasugrel (75 mg, 0.2 mmol) and 22 mg PLE were suspended in 40 mL acetone, 140 mL deionized water and 20 mL KP_i_ buffer (200 mM, pH 7) and stirred at 25 °C and 200 rpm. After 30 min of reaction time, 350 mg ascorbate and 600 U *Mro*UPO (50 mg) were added. Hydrogen peroxide (400 mM) was continuously added via a syringe pump with a flow rate of 1 mL h^−1^ (corresponding to 2 mM h^−1^), and the reaction mixture was stirred for an additional 2.5 h. Samples were taken every 15 min as described above. Then the mixture was extracted three times with dichloromethane. The combined organic phases were dried with sodium sulfate, filtered and evaporated to dryness under vacuum resulting in 54 mg of brownish resin. The crude product was purified by preparative liquid chromatography to give 17 mg of a bright yellow mixture of PAM isomers (24% yield, >95% purity, molar ratio 43:57). HR-ESI-MS: 350.1219 [M + H]^+^; theor. for C_18_H_20_FNO_3_S + H^+^: 350.1226. ^1^H NMR (300 MHz, CDCl_3_) *δ* 7.41–7.24 (m, 2H), 7.20–7.08 (m, 2H), 5.59 (s, 0.4H), 5.51 (s, 0.6H), 5.24 (m, 1H), 4.75 (s, 1H), 3.47 (d, *J* = 12.8 Hz, 0.4H), 3.27 (d, *J* = 12.7 Hz, 0.6H), 3.20 (d, *J* = 12.9 Hz, 0.4H), 3.08 (d, *J* = 12.8 Hz, 0.6H), 2.85 (d, *J* = 11.8 Hz, 0.4H), 2.73 (d, *J* = 12.0 Hz, 0.6H), 2.66 (td, *J* = 11.9, 2.6 Hz, 0.4H), 2.42 (td, *J* = 11.9, 2.5 Hz, 0.6H), 2.32–1.99 (m, 3H), 1.84–1.72 (m, 1H), 1.07–0.95 (m, 2H), 0.91–0.73 (m, 2H). ^13^C NMR (126 MHz, CDCl_3_) *δ* 207.15, 169.73, 162.33, 160.36, 159.19, 130.62, 130.20, 124.54, 121.16, 115.96, 114.29, 71.59, 54.84, 52.63, 46.83, 44.56, 32.82, 31.87, 18.61, 12.11, 11.59.

#### 2.4.4. Preparative Liquid Chromatography

The isolated crude products of the semi-preparative synthesis approaches were purified using PrepHPLC (Shimadzu, Kyoto, Japan) comprised with a CBM-20A system controller, LC-20AP pump system, SPD-10A VP detector (DAD) and fraction collector FRC-10A. The oily crude products were dissolved in 50% (*v*/*v*) acetonitrile and subjected to preparative chromatography under the following conditions: LiChroCART^®^ (LiChrospher 100) C18 column (250 × 10 mm, 10 µm), flow rate 5 mL min^−1^, UV detection at 220 nm, injection volume 500 µL, sample concentration approx. 10 mg mL^−1^; and isocratic conditions: 50% MeCN for CAM and 2-oxo-PSG and 35% MeCN for PAM. The combined fractions were evaporated to remove acetonitrile and finally lyophilized.

### 2.5. Analytical Methods

#### 2.5.1. High-Performance Liquid Chromatography

The HPLC system (VWR Hitachi International GmbH, Darmstadt, Germany) comprised an L-2130 pump, L-2200 autosampler, L-2300 column oven and L-2455 DAD coupled with a low-temperature evaporative light scattering detector (ELSD 100, VWR, Radnor, PA, USA). Separation was performed on a Kinetex^®^ column (C18, 5 µm, 100 Å, 150 × 4.6 mm, Phenomenex, Torrance, CA, USA) with mobile phase A (diH_2_O, 0.1% formic acid) and B (acetonitrile, 0.1% formic acid) using the following gradient at a flow rate of 1 mL min^−1^: 0 min, 20% B; 2 min, 20% B; 13 min, 45% B; 20 min, 80% B; 22 min, 80% B; 23 min, 20% B and 26 min, 20% B. Analytes were identified using authentic standards and high-resolution mass spectrometry (HRMS). In order to quantify the active metabolites, CAM and PAM, the samples isolated during the study were used for their calibration.

#### 2.5.2. High-Resolution Mass Spectrometry

Chromatographic separation for LC-MS experiments were performed on a Thermo Scientific Vanquish Flex Quaternary UHPLC system (Thermo Fisher Scientific, Waltham, MA, USA) using a Kinetex^®^ EVO column (C18, 5 µm, 100 Å, 150 × 4.6 mm, Phenomenex, Torrance, CA, USA). The injection volume was 1 µL, and the column was eluted at a flow rate of 0.5 mL min^−1^ and 40 °C with two mobile phases A (diH_2_O, 0.1% formic acid) and B (acetonitrile, 0.1% formic acid) and following gradient: 0 min, 30% B; 2 min, 30% B; 13 min, 55% B; 14 min, 80% B; 16 min, 80% B; 17 min, 30% B and 20 min, 30% B.

MS and MS^2^ spectra were obtained using a Thermo Scientific Q Exactive Plus Quadrupole-Orbitrap mass spectrometer (Thermo Electron, Waltham, MA, USA) coupled with a heated electrospray ionization source in positive mode. The tune operating parameters were as follows: the rate of sheath gas flow and auxiliary gas flow were 60 and 15 (arbitrary unit), respectively; spray voltage, 4.0 kV; the temperatures of capillary and auxiliary gas heater were 320 and 400 °C, respectively; high-resolution MS was operated at full scan mode with a mass range of *m*/*z* 150–1500 at a resolution of 70,000 (*m*/*z* 200). The MS^2^ data at a resolution of 35,000 were obtained by parallel reaction monitoring mode triggered by inclusion ions list, which was built by the molecule predicted. The collision energy varied between CE10 and CE50 and is indicated with the respective results.

#### 2.5.3. NMR Studies

^1^H- and ^13^C NMR spectra (500 and 126 MHz, respectively) were recorded on a Bruker Advance neo 500 spectrometer (Billerica, MA, USA) in the solvents indicated. Chemical shifts (*δ*) are given as ppm relative to (CH_3_)_4_Si.

## 3. Results

In order to elucidate the feasibility of the oxidation of antithrombotic thienopyridines with UPOs, the studies were first limited to clopidogrel as a substrate because of the two-step oxidation cascade. Incubation of clopidogrel with several UPOs was performed in the presence of an organic co-solvent in a phosphate-buffered system. The oxidant hydrogen peroxide was added intermittently over the reaction period. Ascorbate was added to prevent coupling reactions, which may occur due to the radical-forming peroxidase activity of UPOs [29]. Five homologously produced UPOs (wild-type enzymes) were tested: *Aae*UPO, *Cra*UPO, *Mro*UPO, *Cgl*UPO and *Mwe*UPO (entry 1–7, Table 1). With the two UPOs of the genus *Marasmius* (*Mro*UPO, *Mwe*UPO), the reaction was additionally carried out at pH 5.5 (entry 4 and 7, Table 1), as this pH corresponds to their reaction optimum [34,37].

**Figure 2:**
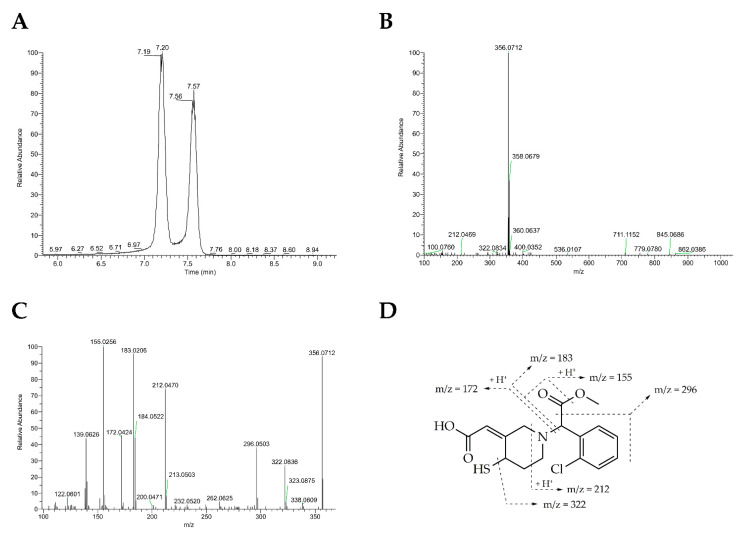
Extracted ion chromatogram and MS and MS^2^ spectra of isomers of active clopidogrel metabolite (CAM) formed during conversion of 1 mM clopidogrel by 2 U mL^−1^ *Mro*UPO in presence of 5 mM ascorbate, 20 mM KP_i_ buffer (pH 7.0) and in sum 4 mM hydrogen peroxide. (**A**) Extracted ion chromatogram at *m*/*z* 356 (MS); (**B**) MS spectra of the two isomers; (**C**) MS^2^ spectra of the ion at *m*/*z* 356 with collision energy of CE20; (**D**) assignment for the fragmentation pattern in Figure 2C.

HPLC-MS studies of the incubated samples revealed the incipient formation of two pairs of metabolites, each at the same ratio and, to some extent, of partially oxygenated byproducts. The dominant pair was identified as 2-oxo-clopidogrel, as could be deduced from the identical MS^2^ spectra that were obtained for the parent ion (*m*/*z* = 338.0612, C_16_H_17_ClNO_3_S^+^) with the associated fragments (*m*/*z* 183.0208 (100%) and *m*/*z* 155.0258 (87%), CE20) when using the appropriate reference standard. The second metabolite pair appears to be a stable dimer of oxygenated clopidogrel, which was not further converted by UPOs (*m*/*z* 675.1150, C_32_H_33_Cl_2_N_2_O_6_S_2_^+^). Experiments with an increased concentration of radical scavengers did not lead to decreased dimer formation (data not shown). The ratio of 2-oxo-CPG and oxo-CPG-dimer was approx. 2:1 in the case of *Mro*UPO and *Mwe*UPO. In contrast, the ratio obtained with *Aae*UPO was about 1:1 and with *Cra*UPO and *Cgl*UPO even 1:3. A third less dominant pair of metabolites exhibited molecular masses of *m*/*z* 356/358 in the isotopic ratio typical for the presence of a chlorine atom. Fragmentation of the parent ion (*m*/*z* 356.0712 (^35^Cl), C_16_H_19_ClNO_4_S^+^, CE20) gave clear indication that these are two diastereomers of CAM (Figure 2). The highest yields under the conditions described above were obtained with the UPOs from *Marasmius* spp., i.e., *Mro*UPO (8.6%) and *Mwe*UPO (6.4%) at pH 7.0 (entries 3 and 6, Table 1). A representative chromatogram with the three metabolite pairs and the MS and MS^2^ spectra of the dimer are given in the Supplementary Material (Figures S1 and S2).

**Figure 3:**
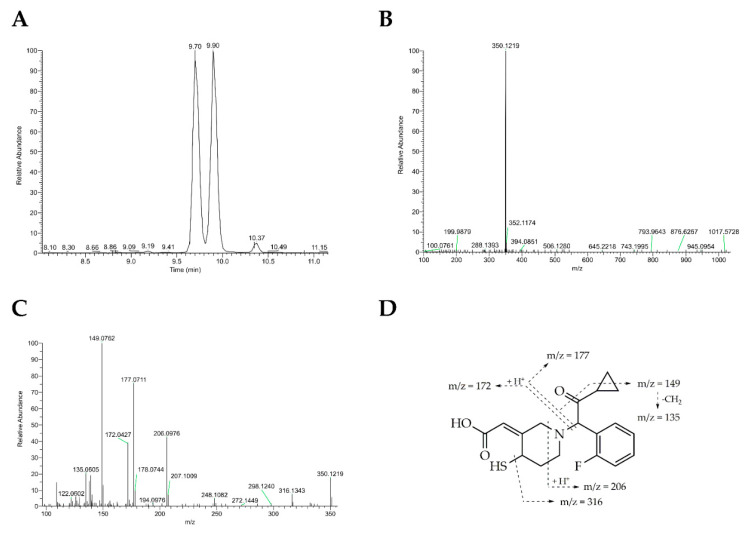
Extracted ion chromatogram and MS and MS^2^ spectra of isomers of active prasugrel metabolite (PAM) formed during conversion of 1 mM prasugrel by 2 U mL^−1^ PLE for 30 min followed by conversion with 2 U mL^−1^ *Mro*UPO in the presence of 5 mM ascorbate, 20 mM KP_i_ buffer (pH 7.0) and in sum 4 mM hydrogen peroxide. (**A**) Extracted ion chromatogram at *m*/*z* 350 (MS); (**B**) MS spectra of the two isomers; (**C**) MS^2^ spectra of the ion at *m*/*z* 350 with collision energy of CE25; (**D**) assignment for the fragmentation pattern in (**C**).

The conversion of prasugrel (PSG) requires hydrolysis of the acetic acid ester in the first step. In order to carry out the entire reaction sequence in one reaction batch, hydrolysis with porcine liver esterase (PLE) was investigated. For this purpose, 1 mM substrate was dissolved in 10% acetone in a phosphate-buffered system (pH 7.0) and incubated with 2 U mL^−1^ PLE. PSG was completely converted within 30 min to 2-oxo-prasugrel, which was also identified as a double peak (ratio 1:1) of two diastereomers (*m*/*z* 332.1115, C_18_H_19_FNO_2_S^+^). Subsequently, ascorbate, UPO and successively hydrogen peroxide was added over a period of 2 h. HPLC-MS analyses of incubated samples revealed that *Mro*UPO (34%, entry 10) and *Mwe*UPO (31%, entry 12) particularly catalyzed the formation of a pair of metabolites, each with *m*/*z* 350 (pair of diastereomers in ratio 1:1). Again, MS (*m*/*z* 350.1219, C_18_H_21_FNO_3_S^+^) and MS^2^ data (from *m*/*z* 350.1219, CE25) gave clear hints for the formation of the active prasugrel metabolite PAM (Figure 3). Incubation with *Cgl*UPO also resulted in a high yield of PAM, but numerous byproducts emerged (entry 11, Table 1).

The diastereomeric pairs are likely due to the configuration of the thiol group at C4 or the *cis*/*trans* configuration at C3. To obtain precise insights into the chemical nature of the products, CAM and PAM (presumably identical to human metabolites), NMR studies were performed. For this, the reactions had to be optimized and upscaled in order to isolate the metabolites.

With the aim of optimizing the reaction conditions and increasing the yield of active metabolites, especially in the case of clopidogrel, both reductant and organic solvents as well as their concentration were varied in the approach with *Mro*UPO (Table 2). It turned out that the presence of a reductant was particularly necessary. The absence of ascorbate or glutathione resulted in a variety of oxidation and coupling products, with only small amounts of the desired compounds being detected with LC-MS. When glutathione replaced ascorbate, the formation of CAM was significantly reduced (entry 14, Table 2), and a mixture of four isomers with the appropriate molecular mass of *m*/*z* 356/358 was identified in traces. Replacing acetone with other water-miscible solvents such as acetonitrile, methanol, dimethyl sulfoxide or dimethyl formamide did not increase the yields of the active metabolite. Interestingly, the reaction with methanol as co-solvent gave a good yield of 2-oxo-CPG (31%) but almost no formation of the active metabolite. Increasing the amount of acetone to 20% increased the CAM yield to about 10%, which was the highest yield that could be achieved (entry 21, Table 2). Further extensive attempts such as exchanging buffer systems, varying the substrate–catalyst ratio or the amount and dosing rate of the co-substrate hydrogen peroxide as well as the use of other hydroperoxides or hydrogen peroxide producing systems (e.g., glucose/glucose oxidase, carbamide peroxide) did not significantly improve the yields (data not shown).

**Figure 4:**
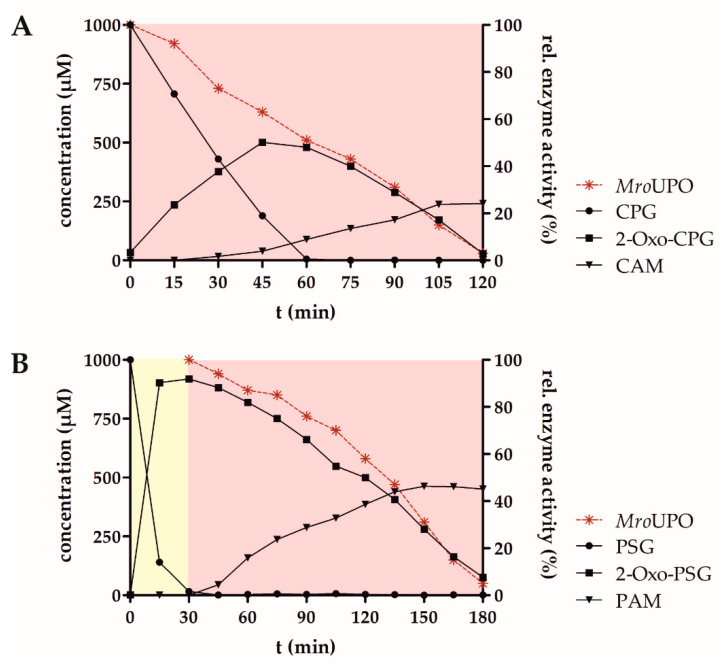
Kinetics of clopidogrel (**A**) and prasugrel (**B**) conversion (0.2 mmol each) by 2 U mL^−1^ *Mro*UPO in presence of 5 mM ascorbate, 20 mM KP_i_ buffer (pH 7.0) and continuous supply of hydrogen peroxide (2 mM h^−1^). Prasugrel was preincubated with 2 U mL^−1^ PLE for 30 min. Black circles are the thienopyridine substrates; black squares represent the 2-oxo metabolites and black triangles are the active metabolites. Isomeric double peaks were merged. The red stars reflect the residual UPO activity. Yellow background represents the PLE reaction; red background indicates the UPO reaction. Ordinates are the concentrations of the metabolites recalculated after calibration with isolated compounds.

The optimal reaction conditions (entry 21, Table 2) were chosen to perform the syntheses of CAM and PAM at a 100 mg scale. The hydrogen peroxide feed was changed from intermittent addition to continuous feed by a syringe pump to ensure a permanent low hydrogen peroxide concentration and thus an optimal UPO performance. During the reaction, the concentrations of substrate, 2-oxo metabolite and active metabolite as well as the residual activity of the catalyst were monitored. Clopidogrel was almost completely converted within one hour, with the formation of 2-oxo-CPG predominantly at the beginning and, to a somewhat minor extent, the previously mentioned dimerization product. After about 45 min, the concentration of 2-oxo-CPG reached a maximum of about 500 µM and declined afterwards as the thiolactone was further converted mostly to the active metabolite (Figure 4A). Here, an isomer of 2-oxo-CPG was preferentially transformed by *Mro*UPO (Figure S1B). After 2 h, the residual activity of *Mro*UPO was below 5%, and 2-oxo-CPG was almost completely converted. The final concentration of active metabolite (pair of stereoisomers) was about 250 µM, and both isomers were present in equal amounts. The metabolite pair was successfully isolated and purified by preparative HPLC. NMR spectra were difficult to interpret because of the presence of isomers. Therefore, spin–spin coupling constants could not be determined in some cases due to the overlapping of the signals of both compounds. The ratio of isomers in the isolate was fortunately about 2:3 so that the individual signals could be assigned to the metabolites with the use of the correlation spectra (COSY, HMBC, HSQC). The singlets of the ^1^H NMR spectra at 5.56 ppm (60%) and 5.45 (40%) were allocated to H-16 and confirmed the *cis* configuration at C3–C16 (CAM-H3 and CAM-H4), which were in agreement with those in the literature [20]. The metabolite, which was initially formed and preferably by *Mro*UPO, eluted earlier from the HPLC column and was identified as CAM-H3. In principle, the isolation of 2-oxo-CPG would also be possible by a kinetically controlled approach. However, the isolation of the metabolites will be challenging due to the presence of the oxo-CPG dimer.

Incubation of 150 mg prasugrel with PLE at 25 °C and pH 7 resulted in complete conversion after 60 min as verified by TLC. MS and MS^2^ data confirmed the exclusive formation of 2-oxo-clopidogrel. For structural confirmation, the metabolite pair was isolated by preparative HPLC. In a second batch, 75 mg prasugrel was incubated analogously, with a complete turnover after 30 min as analyzed by TLC and HPLC. After subsequent addition of *Mro*UPO, reductant and feeding of hydrogen peroxide, 2-oxo-prasugrel was continuously converted in an almost linear way to the stereoisomer pair PAM (Figure 4B). In this case, no preferential conversion of an isomer by UPO was observed. After 180 min total reaction time, the yield of PAM was about 44%. NMR analyses confirmed the formation of the active metabolites in *cis* configuration analogously to CAM. HPLC-ELSD elution profiles, ^1^H and ^13^C NMR spectra as well as two-dimensional correlation spectra (COSY, HMBC, HSQC) of isolated CAM, PAM and 2-oxo-PSG as well as the assignment of the signals are given in Supplementary Material.

## 4. Discussion

**Scheme 2:**
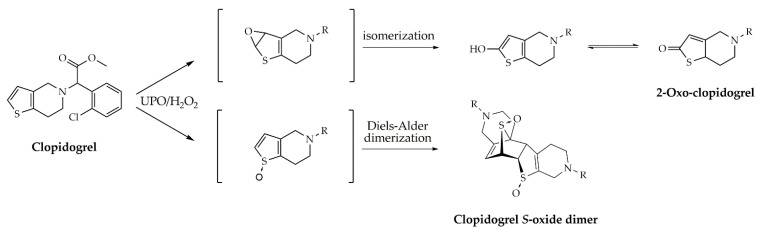
Proposed mechanism for product formation in the UPO-catalyzed oxygenation of clopidogrel.

The aforementioned results show that UPOs can be used for efficient syntheses of active thienopyridine metabolites. The crucial step in the case of clopidogrel seems to be the formation of the 2-oxo metabolite. Analysis of the reaction kinetics revealed that a pair of isomers was formed in parallel with 2-oxo-CPG, which may be a dimer of oxygenated CPG based on the molar mass. Such dimer formation has not been described in the metabolism of CPG by P450s. However, in the reaction described here, these dimers represent about 30% of the products formed (using *Mro*UPO) and, thus, significantly reduce the final yield of CAM. Since the focus of this study was on the syntheses of CAM and PAM, the dimer mixture was not isolated and further analyzed by NMR. Nevertheless, the formation of a dimer of CPG *S*-oxide can be assumed. The oxidation of aromatic rings by both P450s and UPOs is thought to proceed not via H-atom abstraction (as in the case of the hydroxylation of sp^3^-carbons), but via the formation of arene oxides (epoxides), which subsequently rearrange under an NIH shift to phenols [38,39,40]. Similar arene oxide formation has been described for the oxygenation of thiophenes resulting in thiophen-2-ones, e.g., in the case of CPG metabolism. As an alternative to arene oxide formation, UPO-catalyzed oxygenation may also occur at the heteroatoms to form the corresponding *S*-oxides [27,41]. Thiophene-*S*-oxides can undergo Diels–Alder reaction to form stable dimers [42,43,44]. Such a reaction type is also conceivable with clopidogrel and reflects the distribution between aromatic hydroxylation and *S*-oxidation in the first reaction step (Scheme 2).

This also provides a plausible explanation for the different distribution of 2-oxo-CPG and CPG *S*-oxide dimers when using different UPOs. A product shift to the 2-oxo metabolite might be achieved by protein engineering. Such attempts have resulted in improved UPO variants ensuring higher yields, for example, in the synthesis of 5-hydroxypropranolol and dextromethorphan [32,45]. In our context, it would be advisable to optimize UPOs by directed evolution both toward arene oxide formation and *S*-oxidation. In this way, a two-step reaction setup with optimal yields could be established. According to our results, *Aae*UPO would be a good candidate for arene oxide formation (epoxidation), since the distribution was about 1:1, and *S*-oxidation of 2-oxo-CPG was not observed. *Cgl*UPO, on the other hand, would be a good candidate for improving *S*-oxidation, which was shown in the reactions with 2-oxo-PSG. However, the best turnover in the two-step reaction with the wild-type enzymes was obtained with *Mro*UPO in this study. The continuous loss of activity of *Mro*UPO that was observed during the reaction is a known phenomenon for some heme-containing enzymes and might be caused by heme bleaching in consequence of increasing peroxide concentration [46].

UPOs’ ability to oxygenate heteroatoms is particularly evident in the second reaction step. The *S*-oxidation desired constitutes the ‘triggering sequence’ for ring opening to form the active metabolite. Over the years, Dansette et al. have conducted extensive studies on the metabolism of clopidogrel, partly adapted it to prasugrel and elucidated the intermediates of the complex mechanism as well as of influences on the position of the double bonds [7,8,9,10,12,47]. The effects of ascorbate and glutathione observed in experiments with human liver microsomes (HLMs) were the same as with UPOs in this study. In the presence of ascorbate, only the *cis* thiol metabolites were formed, whereas *cis* and *trans* thiol metabolites emerged in the presence of glutathione as reductant [47]. Given the very similar behavior of clopidogrel and prasugrel in the UPO reactions described above, and in comparison to the P450 reactions in HLMs reported in the literature, this example demonstrates again that UPOs can make a crucial contribution to the elucidation of human biotransformation processes. The reaction setups are much easier to perform, less expensive and linearly scalable. Of course, they cannot replace studies with human or animal cell material, but for the synthesis of metabolites as reference material or to support structural elucidation, UPOs can be a useful, primarily time-saving tool. It is worth mentioning that only a handful of UPO proteins are available to date, but thousands of putative UPO protein sequences have been identified in genomes widely distributed in the fungal kingdom, that bear an infinite pool of catalytically diverse oxyfunctionalizing biocatalysts [28].

The large-scale application of peroxygenases still needs to overcome a number of bottlenecks, especially regarding their availability. The heterologous production of UPOs has proven to be difficult and has so far only been (really) successful with satisfactory titers for r*Aae*UPO expressed in *Saccharomyces cerevisiae* and *Pichia pastoris* [48]. This expression system has also been used to create numerous interesting mutants [32,45,49,50]. Successful expression of some UPOs was also achieved by Novozymes A/S using *Aspergillus oryzae* as the expression host. These enzymes (r*Cci*UPO, r*Hin*UPO) were tested for several applications, but the exact scales and titers are unknown [33,51,52,53]. Recently, the expression of UPOs in *Escherichia coli* has been successful at small scale, which allowed the generation of purposefully optimized mutants [54,55,56]. However, large-scale UPO production is still limited to r*Aae*UPO, so far [57].

Reaction technology challenges such as optimal peroxide delivery (where sensor-coupled pump systems will be the most elegant solution), stability in the presence of organic solvents and enzyme reusability (immobilization) have also been addressed in several studies but solutions for large-scale syntheses are still missing [27]. Nevertheless, the findings of the last two decades, especially the versatility in the modification of bio-based raw materials (e.g., fats, terpenes, steroids) [58], conversion of environmental pollutants (e.g., PAHs) [59] and synthesis of drug metabolites [26] have shown that the way is open for the application of UPOs in the small-scale chemical segment, such as syntheses of specialty chemicals, sensor technology or for the elucidation of metabolite structures in the pharmaceutical sector.

## 5. Conclusions

We have demonstrated a biocatalytic method for the syntheses of the active metabolites CAM (25% yield) and PAM (44% yield) starting from the parental drugs clopidogrel and prasugrel by using fungal unspecific peroxygenases (UPOs). Both metabolites were isolated and their structure elucidated by mass spectrometry and NMR spectroscopy. This provides easy access to these compounds to be used, e.g., in pharmacological assays or as reference material. Moreover, this UPO-based reaction sequence, starting with the formation of a thiolactone and followed by the cleavage of the thieno ring via a thiolactone sulfoxide intermediate, has been described for the first time and can be adapted to other chemical problems. In general, UPOs could contribute to the elucidation of biotransformation mechanisms catalyzed by hepatic cytochrome P450 enzymes, as there are numerous examples of mimicking intrinsic P450 activity. On the other hand, UPOs can become valuable catalysts for the synthesis of active metabolites of prodrugs for those cases where polymorphism or other deficiencies do not allow a targeted in vivo transformation and personalized therapeutic solutions are required.

## Figures and Tables

**Table 1:** Screening of different UPOs for their ability to convert thienopyridines into 2-oxo metabolites and active metabolites ^1^.

Entry	Substrate	Enzyme(s)	pH	2-Oxo Metabolite (%) ^2^	Active Metabolite (%) ^2^
1	CPG	*Aae*UPO	7.0	29.0 ± 0.5	<2
2	CPG	*Cra*UPO	7.0	12.7 ± 0.3	<2
3	CPG	*Mro*UPO	7.0	10.6 ± 0.2	8.6 ± 0.2
4	CPG	*Mro*UPO	5.5	46.6 ± 0.5	3.6 ± 0.2
5	CPG	*Cgl*UPO	7.0	3.7 ± 0.1	<2
6	CPG	*Mwe*UPO	7.0	8.1 ± 0.9	6.4 ± 0.1
7	CPG	*Mwe*UPO	5.5	31.6 ± 1.2	<2
8	PSG	PLE/*Aae*UPO	7.0	87.4 ± 3.0	5.8 ± 0.6
9	PSG	PLE/*Cra*UPO	7.0	87.2 ± 0.4	<2
10	PSG	PLE/*Mro*UPO	7.0	39.1 ± 2.0	34.2 ± 0.4
11	PSG	PLE/*Cgl*UPO	7.0	19.0 ± 0.5	28.2 ± 1.2
12	PSG	PLE/*Mwe*UPO	7.0	46.5 ± 2.7	31.0 ± 0.3

^1^ The reactions were carried out using 1 mM clopidogrel (CPG) or prasugrel (PSG), 10% acetone, 20 mM KP_i_ buffer, 5 mM ascorbate and 2 U mL^−1^ UPO. Hydrogen peroxide was added every 15 min over a period of 2 h to give a final concentration of 4 mM. In case of PSG, the substrate was preincubated with 2 U mL^−1^ porcine liver esterase (PLE) for 30 min (N = 2). ^2^ Determined by HPLC.

**Table 2:** Optimization of reaction conditions using different solvents and reductants for the conversion of clopidogrel (CPG) by *Mro*UPO ^1^.

Entry	Substrate	Solvent	Reductant	2-Oxo Metabolite (%) ^2^	Active Metabolite (%) ^2^
13 (3)	CPG	Buffer/acetone 90:10	Ascorbate	10.6 ± 0.2	8.6 ± 0.2
14	CPG	Buffer/acetone 90:10	Glutathione	26.2 ± 1.9	<2
15	CPG	Buffer/acetone 90:10	None	<2	<2
16	CPG	Buffer/MeCN ^3^ 90:10	Ascorbate	3.2 ± 0.1	4.0 ± 0.1
17	CPG	Buffer/MeOH ^3^ 90:10	Ascorbate	31.1 ± 0.3	<2
18	CPG	Buffer/DMSO ^3^ 90:10	Ascorbate	16.9 ± 1.8	<2
19	CPG	Buffer/DMF ^3^ 90:10	Ascorbate	25.5 ± 0.5	4.5 ± 0.1
20	CPG	Buffer	Ascorbate	11.5 ± 0.2	<2
21	CPG	Buffer/acetone 80:20	Ascorbate	8.7 ± 0.6	9.8 ± 0.5
22	CPG	Buffer/acetone 60:40	Ascorbate	6.3 ± 0.4	<2

^1^ The reactions were carried out using 1 mM clopidogrel, 20 mM KP_i_ buffer (pH 7), 5 mM reductant and 2 U mL^−1^ *Mro*UPO. Hydrogen peroxide was added every 15 min over a period of 2 h to give a final concentration of 4 mM. ^2^ Determined by HPLC. ^3^ MeCN, acetonitrile; MeOH, methanol; DMSO, dimethyl sulfoxide; DMF, dimethyl formamide.

## Data Availability

The strains and data presented in this study are available on request from the corresponding author.

## Supplementary Materials

The following are available online at https://www.mdpi.com/article/10.3390/jof7090752/s1, Figure S1: LC-MS chromatogram (Full MS) of clopidogrel conversion, Figure S2: MS^1^ and MS^2^ spectra of CPG *S*-oxide dimer, Figure S3: HPLC-ELSD chromatogram of isolated isomers of clopidogrel active metabolite (CAM), Figure S4: ^1^H NMR spectrum of isolated isomers of clopidogrel active metabolite (CAM), Figure S5: ^13^C NMR spectrum of isolated isomers of clopidogrel active metabolite (CAM), Figure S6: COSY spectrum of isolated isomers of clopidogrel active metabolite (CAM), Figure S7: HMBC spectrum of isolated isomers of clopidogrel active metabolite (CAM), Figure S8: HSQC spectrum of isolated isomers of clopidogrel active metabolite (CAM), Figure S9: HPLC-ELSD chromatogram of isolated isomers of 2-oxo-prasugrel, Figure S10: ^1^H NMR spectrum of isolated isomers of 2-oxo-prasugrel, Figure S11: ^13^C NMR spectrum of isolated isomers of 2-oxo-prasugrel, Figure S12: COSY spectrum of isolated isomers of 2-oxo-prasugrel, Figure S13: HMBC spectrum of isolated isomers of 2-oxo-prasugrel, Figure S14: HSQC spectrum of isolated isomers of 2-oxo-prasugrel, Figure S15: HPLC-ELSD chromatogram of isolated isomers of prasugrel active metabolite (PAM), Figure S16: ^1^H NMR spectrum of isolated isomers of prasugrel active metabolite (PAM), Figure S17: ^13^C NMR spectrum of isolated isomers of prasugrel active metabolite (PAM), Figure S18: COSY spectrum of isolated isomers of prasugrel active metabolite (PAM), Figure S19: HMBC spectrum of isolated isomers of prasugrel active metabolite (PAM), Figure S20: HSQC spectrum of isolated isomers of prasugrel active metabolite (PAM), Table S1: Assignment of ^1^H and ^13^C NMR signals to the isomers of clopidogrel active metabolite (CAM), Table S2: Assignment of ^1^H and ^13^C NMR signals to the isomers of 2-oxo-prasugrel, Table S3: Assignment of ^1^H and ^13^C NMR signals to the isomers of prasugrel active metabolite (PAM).
