# Supplementary material for: Biocatalytic Syntheses of Antiplatelet Metabolites of the Thienopyridines Clopidogrel and Prasugrel Using Fungal Peroxygenases
Source: J Fungi (Basel). 2021 Sep 13;7(9):752. doi: 10.3390/jof7090752 (PMC8470877; doi:10.3390/jof7090752)
Supplement: Supplementary file 1 [file jof-07-00752-s001.zip › jof-1377262-supplementary.pdf]

Journal of Fungi

MDPI

Supplementary Materials

Biocatalytic Syntheses of Antiplatelet Metabolites of the Thienopyridines Clopidogrel and Prasugrel Using Fungal Peroxygenases

Jan Kiebist [1,2,*], Kai-Uwe Schmidtke [1], Marina Schramm [1], Rosalie König [1], Stephan Quint [3], Johannes Kohlmann [3], Ralf Zuhse [3], René Ullrich [4], Martin Hofrichter [4] and Katrin Scheibner [1]

[1] Institute of Biotechnology, Brandenburg University of Technology Cottbus-Senftenberg, Universitätsplatz 1, 01968 Senftenberg, Germany
[2] Fraunhofer Institute for Cell Therapy and Immunology, Branch Bioanalytics and Bioprocesses, Am Mühlenberg 13, 14476 Potsdam-Golm, Germany
[3] Chiracon GmbH, Im Biotechnologiepark 9, 14943 Luckenwalde, Germany
[4] Department of Bio- and Environmental Sciences, TU Dresden - International Institute Zittau, Markt 23, 02763 Zittau, Germany
* Correspondence: jan.kiebist@izi-bb.fraunhofer.de

Table of contents

Figure S1. LC-MS chromatogram (FullMS) of clopidogrel conversion 2
Figure S2. MS^1 and MS^2 spectra of CPG *S*-oxide dimer 3
Figure S3. HPLC-ELSD chromatogram of isolated isomers of clopidogrel active metabolite (CAM) 4
Figure S4. ^{1}H NMR spectrum of isolated isomers of clopidogrel active metabolite (CAM) 5
Figure S5. ^{13}C NMR spectrum of isolated isomers of clopidogrel active metabolite (CAM) 6
Figure S6. COSY spectrum of isolated isomers of clopidogrel active metabolite (CAM) 7
Figure S7. HMBC spectrum of isolated isomers of clopidogrel active metabolite (CAM) 8
Figure S8. HSQC spectrum of isolated isomers of clopidogrel active metabolite (CAM) 9
Table S1. Assignment of ^{1}H and ^{13}C NMR signals to the isomers of clopidogrel active metabolite (CAM) 10
Figure S9. HPLC-ELSD chromatogram of isolated isomers of 2-oxo-prasugrel 11
Figure S10. ^{1}H NMR spectrum of isolated isomers of 2-oxo-prasugrel 12
Figure S11. ^{13}C NMR spectrum of isolated isomers of 2-oxo-prasugrel 13
Figure S12. COSY spectrum of isolated isomers of 2-oxo-prasugrel 14
Figure S13. HMBC spectrum of isolated isomers of 2-oxo-prasugrel 15
Figure S14. HSQC spectrum of isolated isomers of 2-oxo-prasugrel 16
Table S2. Assignment of ^{1}H and ^{13}C NMR signals to the isomers of 2-oxo-prasugrel 17
Figure S15. HPLC-ELSD chromatogram of isolated isomers of prasugrel active metabolite (PAM) 18
Figure S16. ^{1}H NMR spectrum of isolated isomers of prasugrel active metabolite (PAM) 19
Figure S17. ^{13}C NMR spectrum of isolated isomers of prasugrel active metabolite (PAM) 20
Figure S18. COSY spectrum of isolated isomers of prasugrel active metabolite (PAM) 21
Figure S19. HMBC spectrum of isolated isomers of prasugrel active metabolite (PAM) 22
Figure S20. HSQC spectrum of isolated isomers of prasugrel active metabolite (PAM) 23
Table S3. Assignment of ^{1}H and ^{13}C NMR signals to the isomers of prasugrel active metabolite (PAM) 24

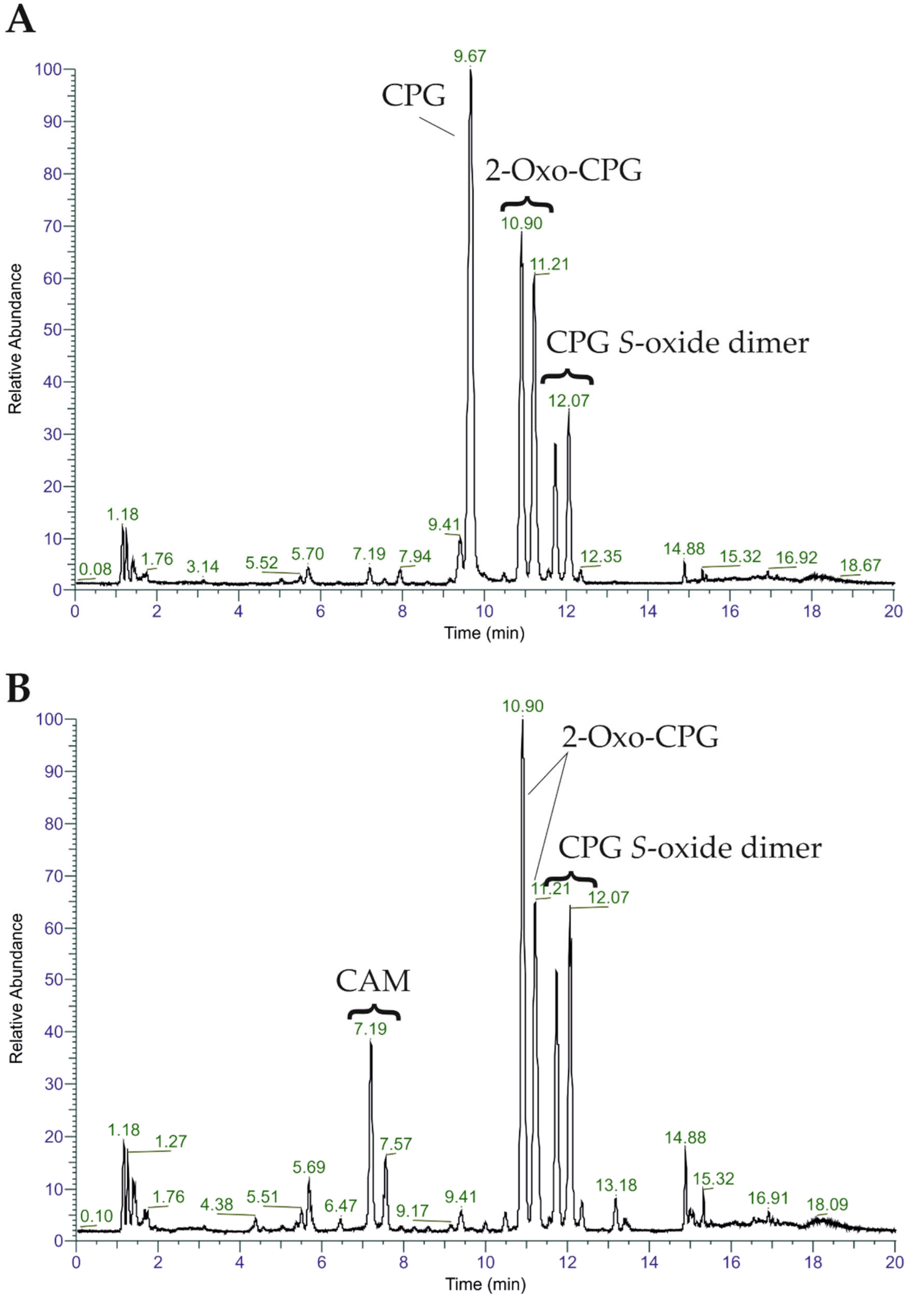

Figure S1. LC-MS chromatogram (FullMS) of clopidogrel conversion with *Mro*UPO after 30 min (A) and 75 min (B).

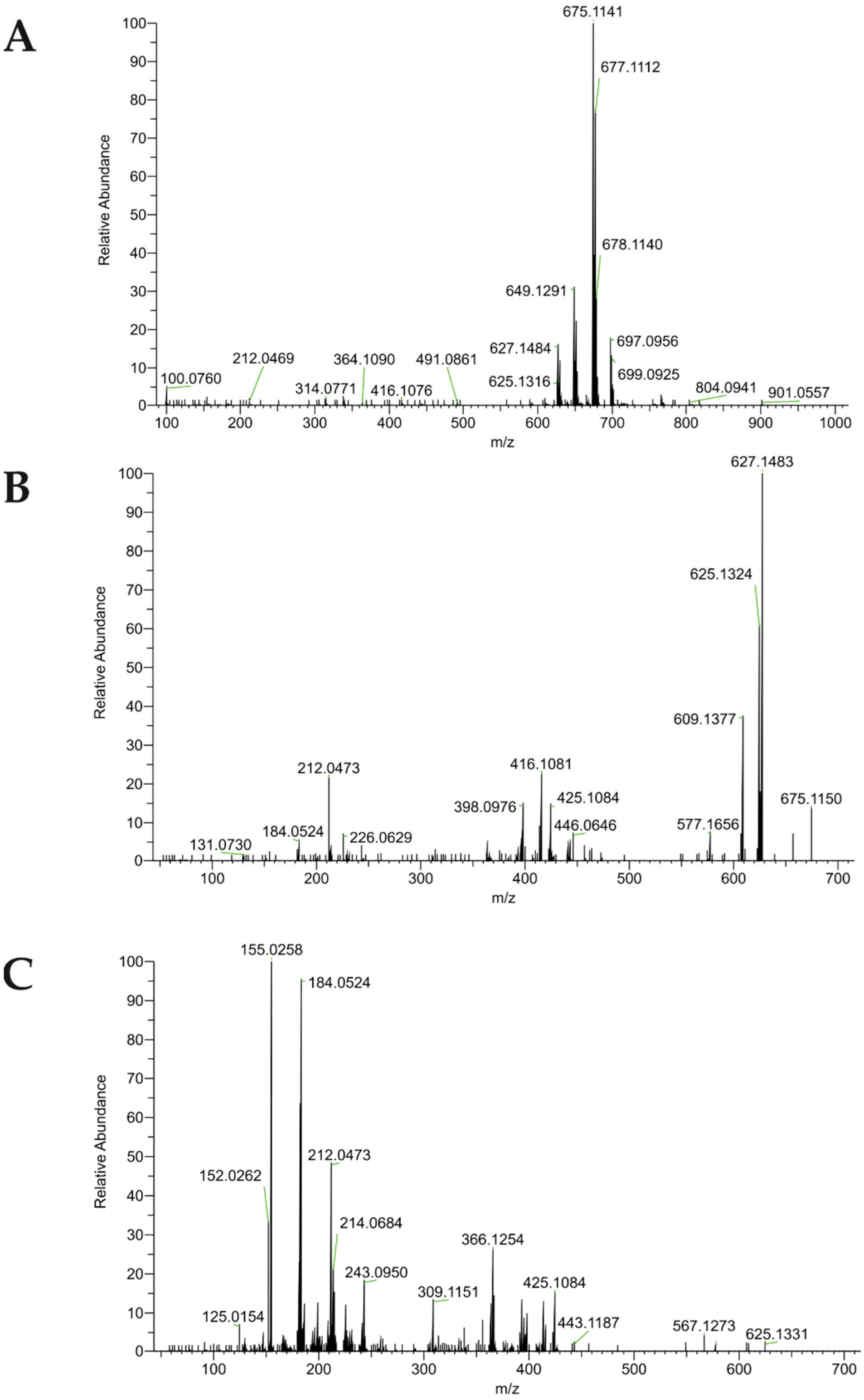

Figure S2. MS[1] and MS[2] spectra of CPG *S*-oxide dimer. (A) MS[1] spectra (B) MS[2] spectra of $C_{32}H_{33}Cl_2N_2O_6S_2^+$ (*m/z* 675.1152) with collision energy CE25 and (C) MS[2] spectra of $C_{32}H_{33}Cl_2N_2O_6S_2^+$ (*m/z* 675.1152) with collision energy CE50.

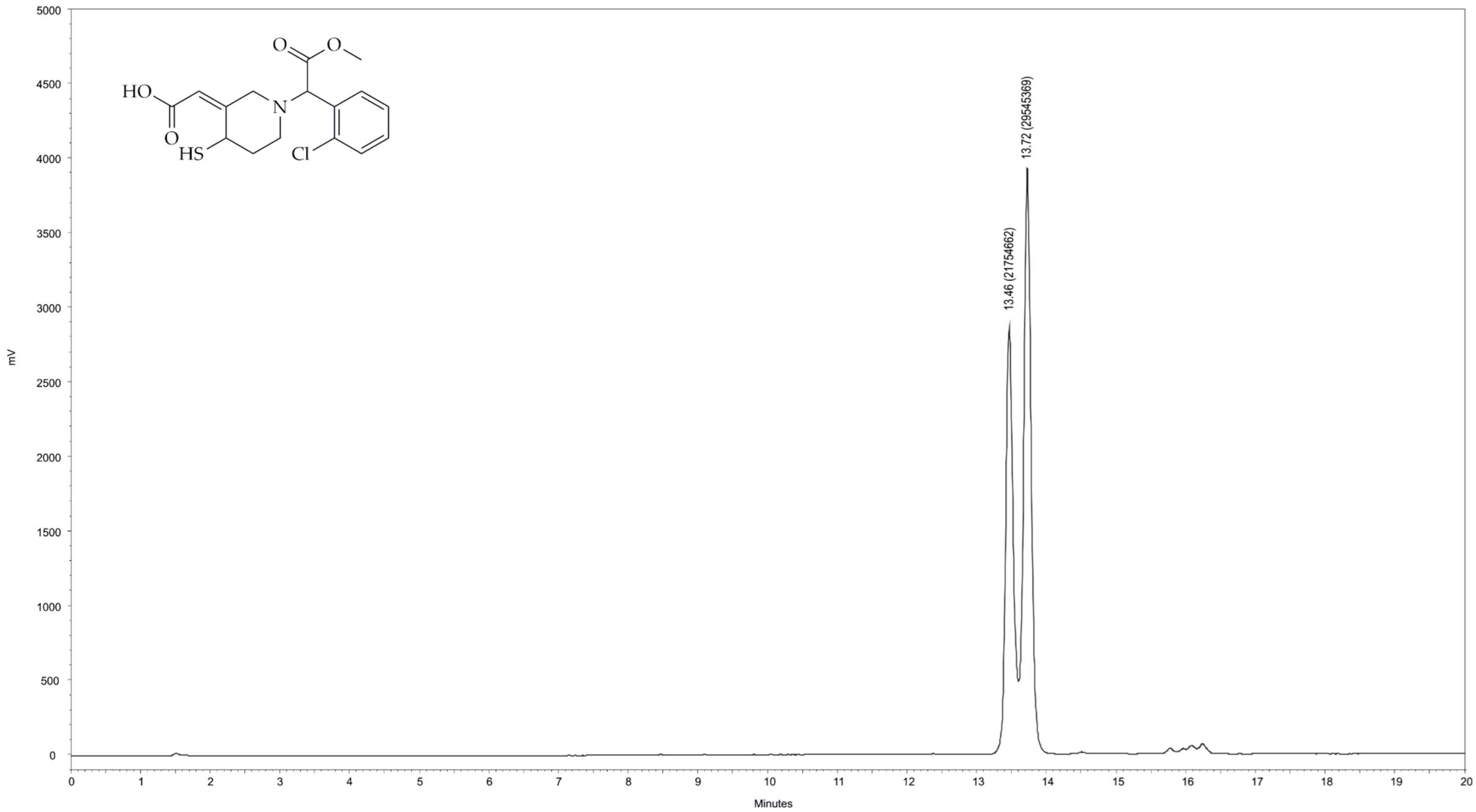

Figure S3. HPLC-ELSD chromatogram of isolated isomers of clopidogrel active metabolite (CAM).

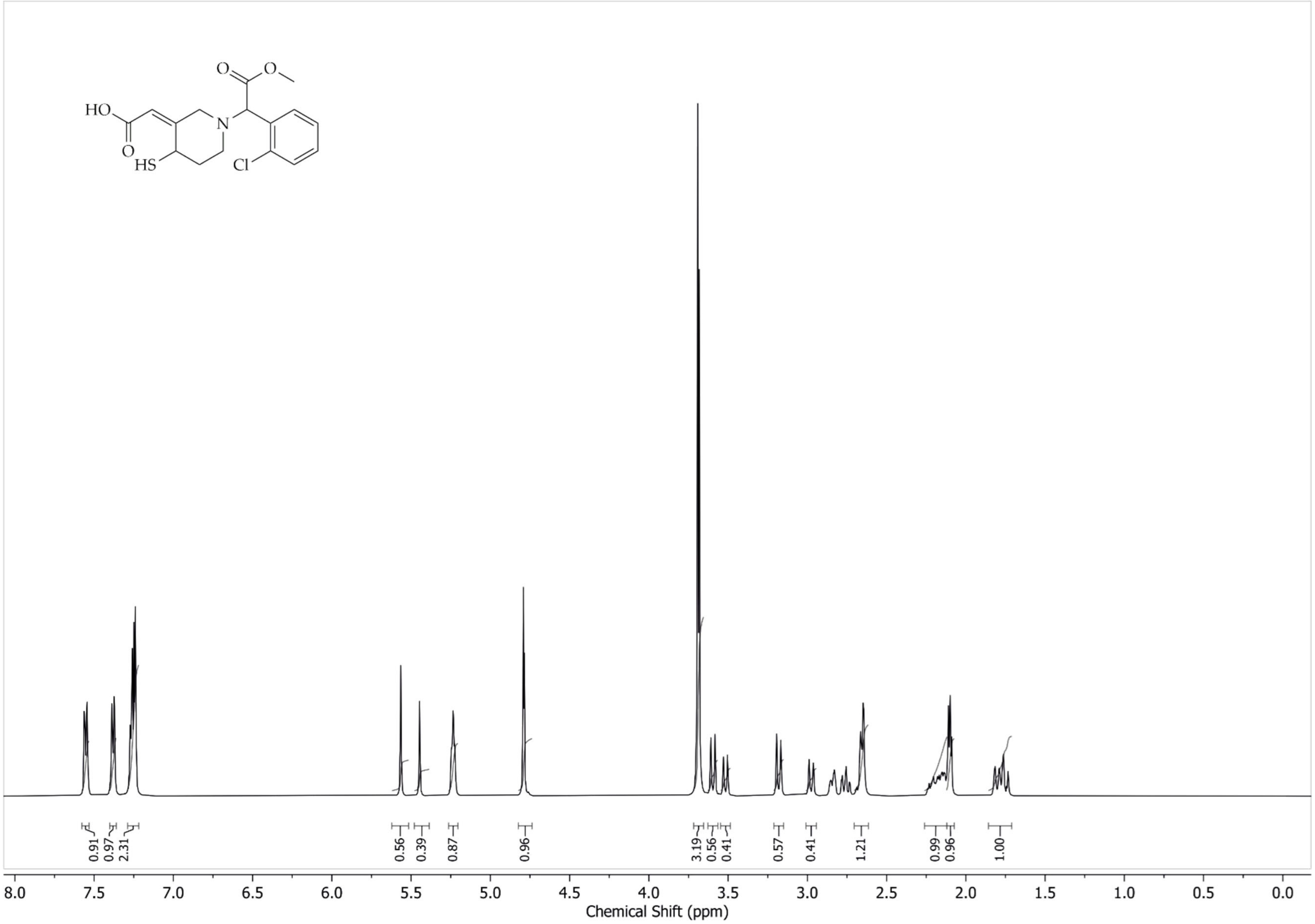

Figure S4. ^{1}H NMR spectrum of isolated isomers of clopidogrel active metabolite (CAM).

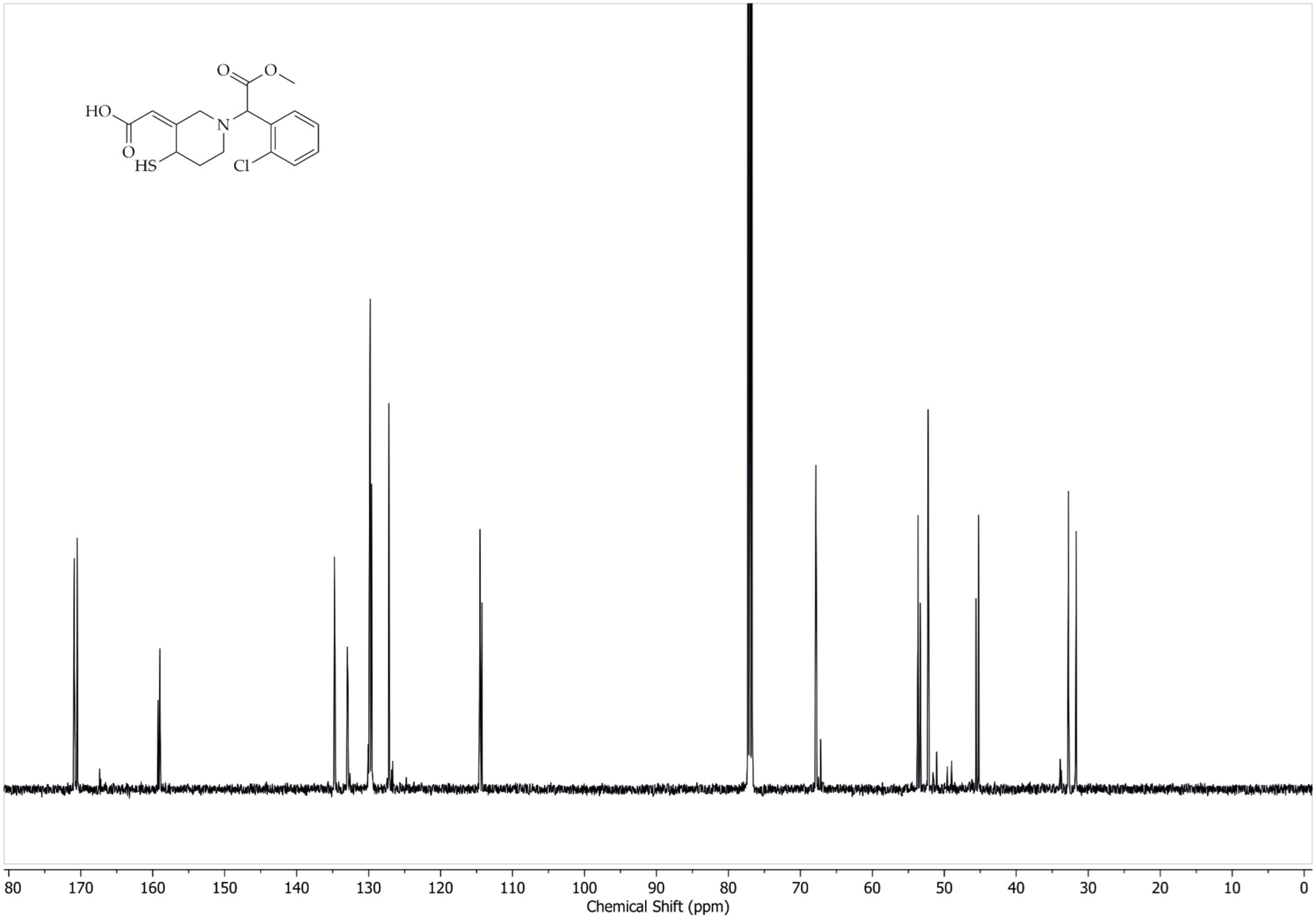

Figure S5. ^{13}C NMR spectrum of isolated isomers of clopidogrel active metabolite (CAM).

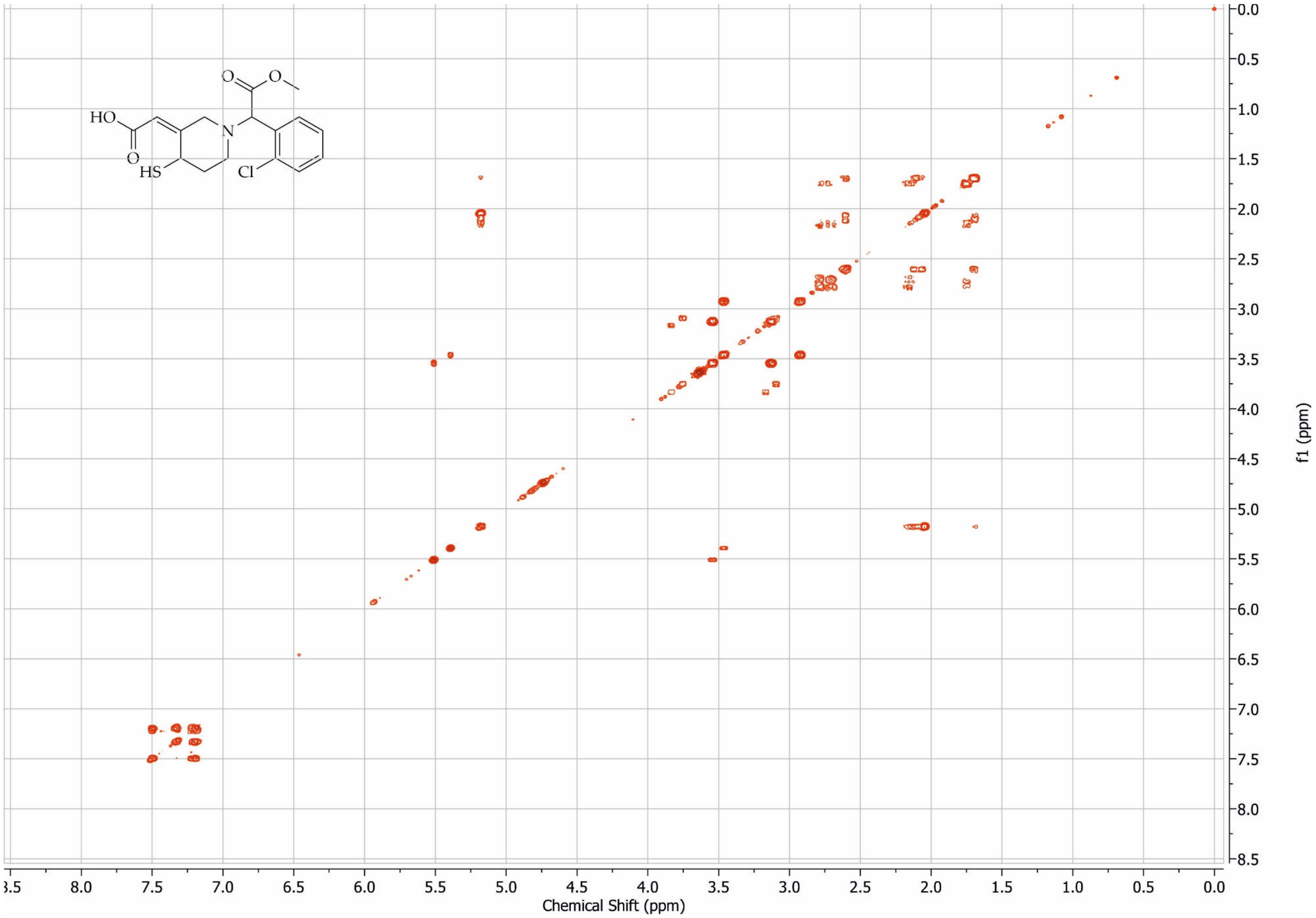

Figure S6. COSY spectrum of isolated isomers of clopidogrel active metabolite (CAM).

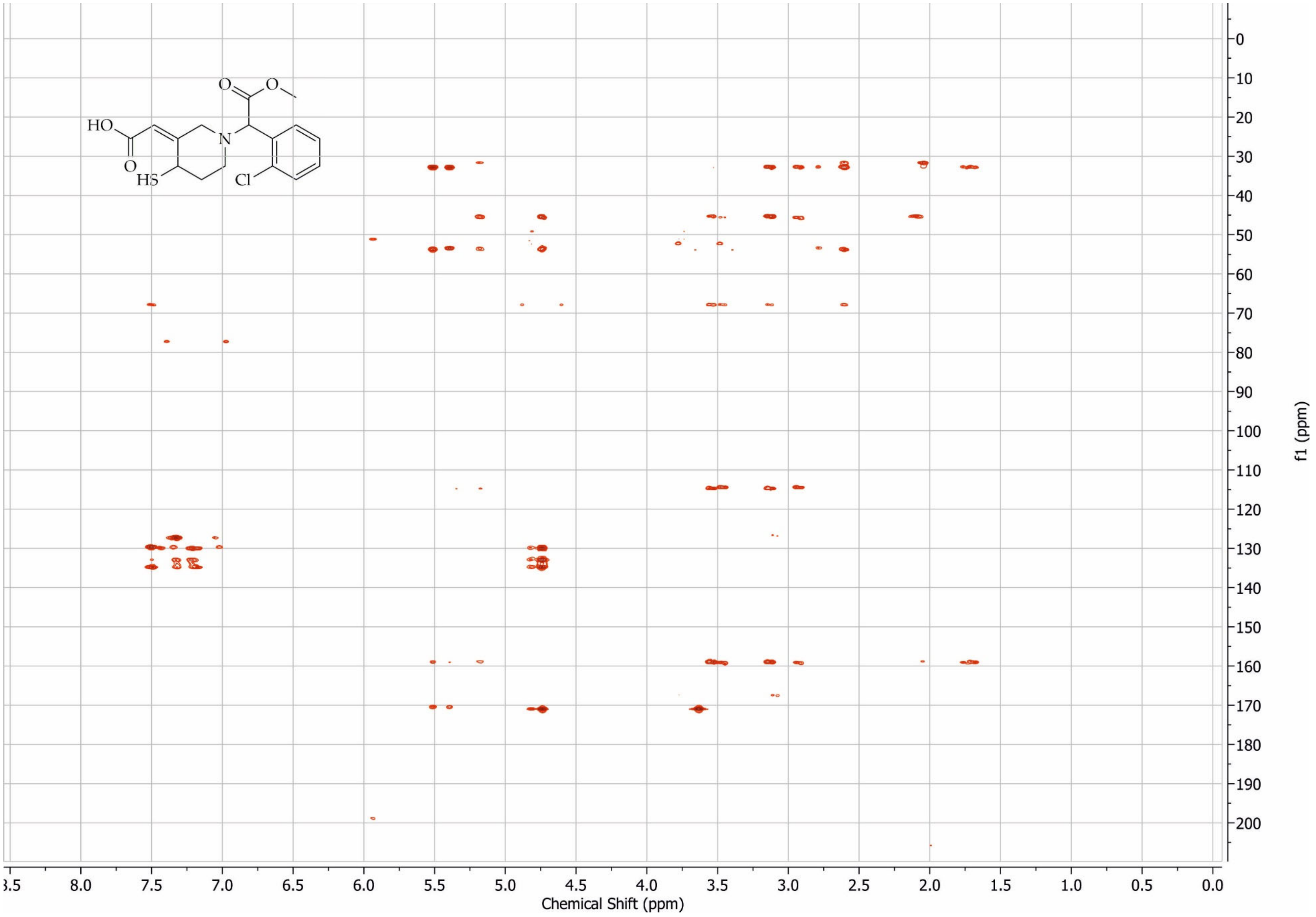

Figure S7. HMBC spectrum of isolated isomers of clopidogrel active metabolite (CAM).

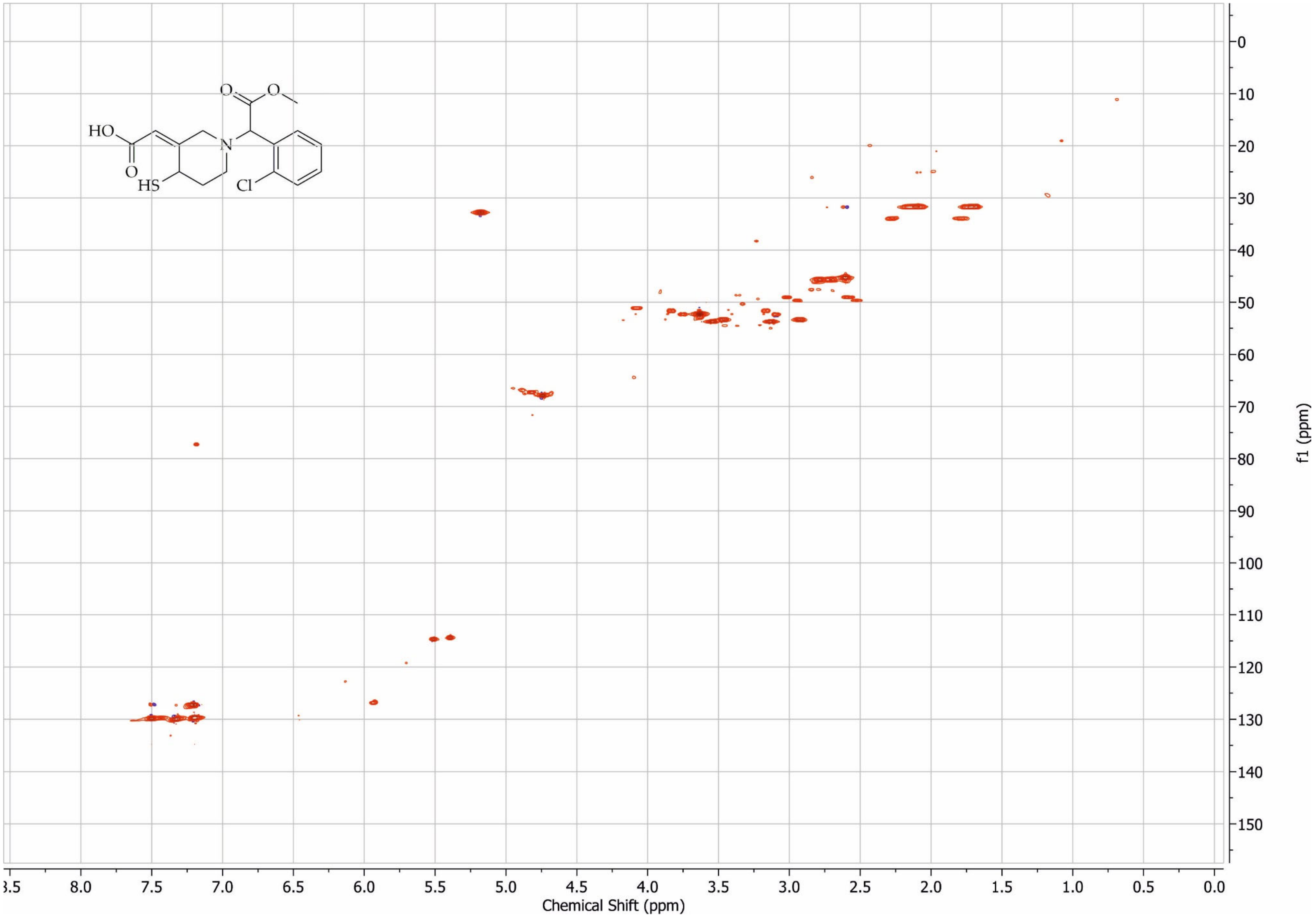

Figure S8. HSQC spectrum of isolated isomers of clopidogrel active metabolite (CAM).

Table S1. Assignment of ^{1}H and ^{13}C NMR signals to the isomers of clopidogrel active metabolite (CAM).

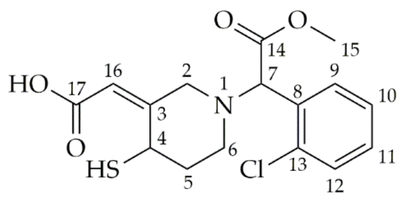

Carbon	δ ^{1}H CDCl$_3$ Isomer a (H4)	δ ^{13}C CDCl$_3$ Isomer a (H4)	δ ^{1}H CDCl$_3$ Isomer b (H3)	δ ^{13}C CDCl$_3$ Isomer b (H3)
2	3.60 ; 3.18	53.67	3.52 ; 2.98	53.35
3	-	159.00	-	159.26
4	5.23	32.75	5.23	32.78
5	2.15 ; 1.75	31.68	2.20 ; 1.80	31.74
6	2.66 ; 2.65	45.24	2.84 ; 2.76	45.59
7	4.79	67.84	4.79	67.80
8	-	134.67	-	134.75
9	7.38	129.89	7.38	130.07
10	7.26	127.17	7.26	127.21
11	7.24	129.58	7.24	129.61
12	7.55	129.74	7.55	129.74
13	-	132.95	-	132.84
14	-	170.90	-	170.94
15	3.69	52.26	3.69	52.21
16	5.56	114.53	5.45	114.24
17	-	170.49	-	170.49
-SH	2.10	-	2.10	-

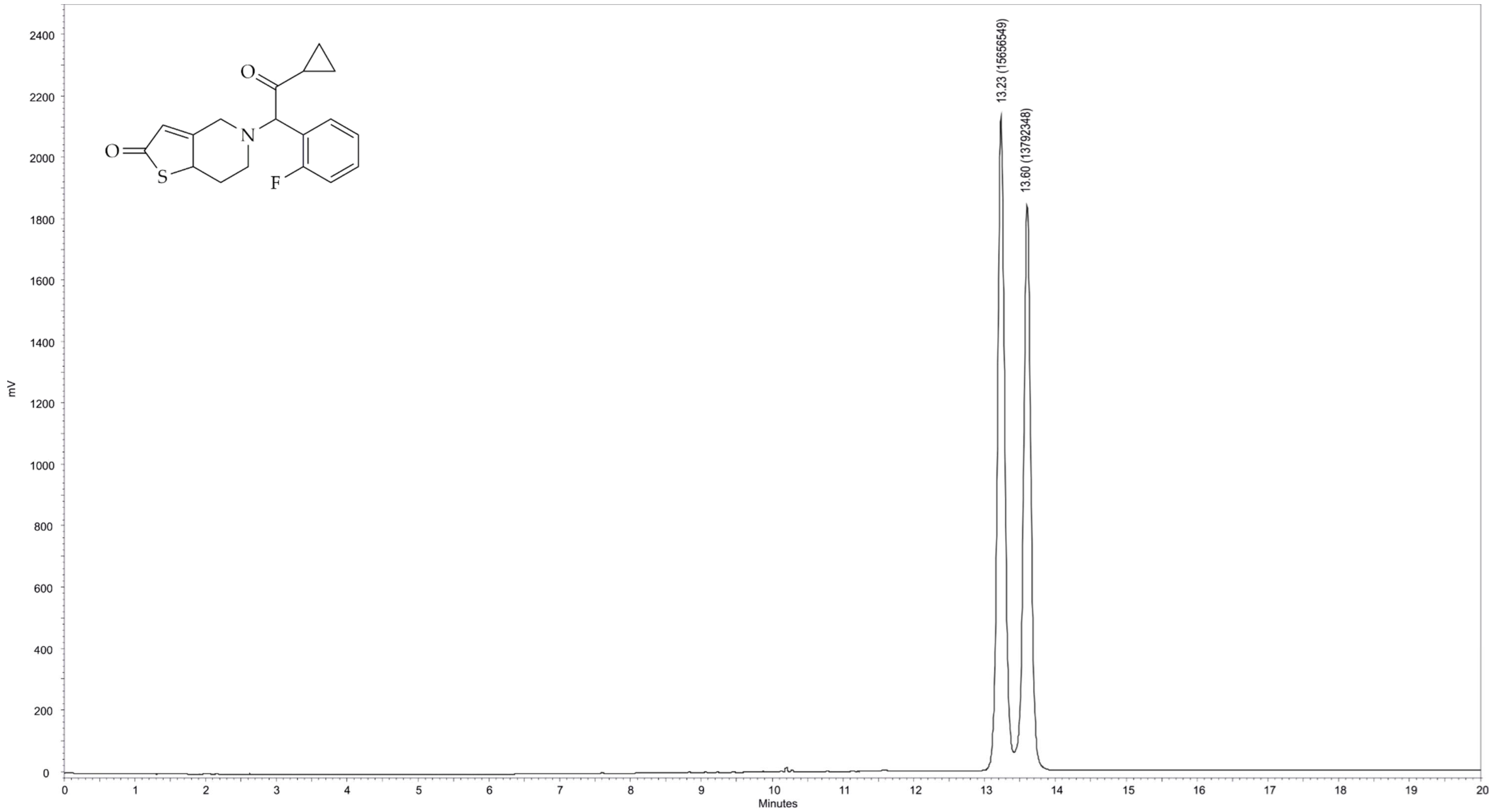

Figure S9. HPLC-ELSD chromatogram of isolated isomers of 2-oxo-prasugrel.

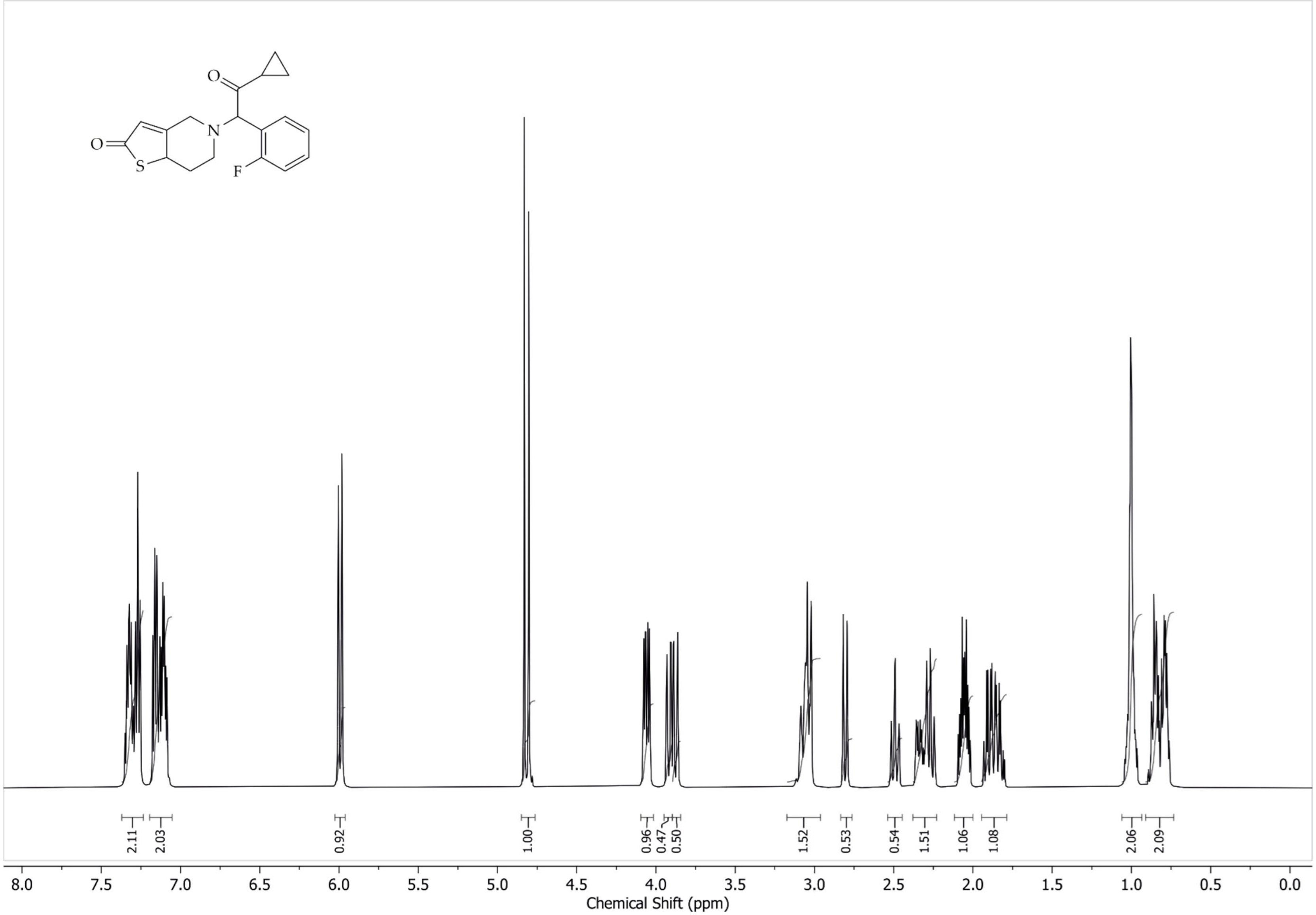

Figure S10. ^{1}H NMR spectrum of isolated isomers of 2-oxo-prasugrel.

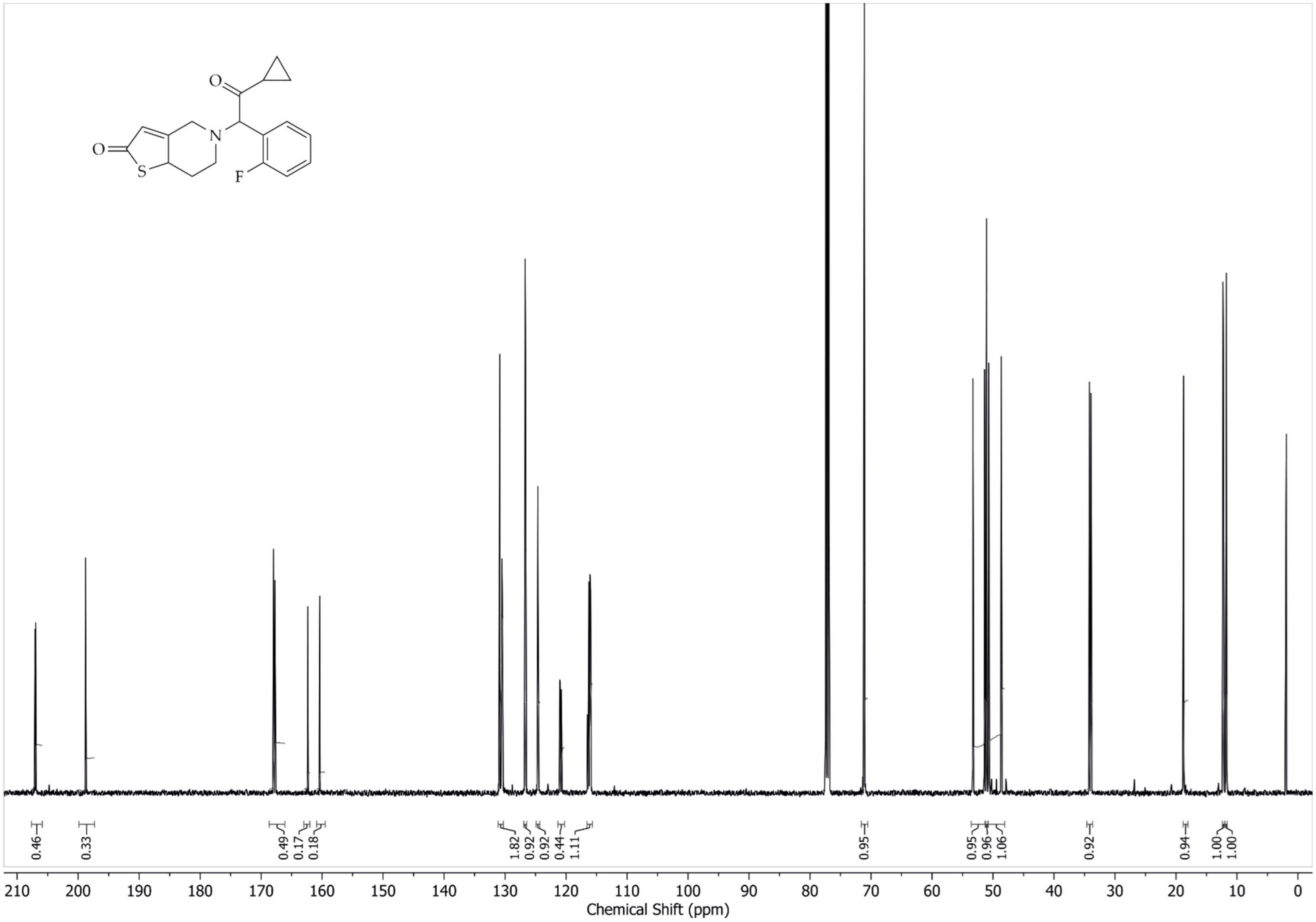

Figure S11. ^{13}C NMR spectrum of isolated isomers of 2-oxo-prasugrel.

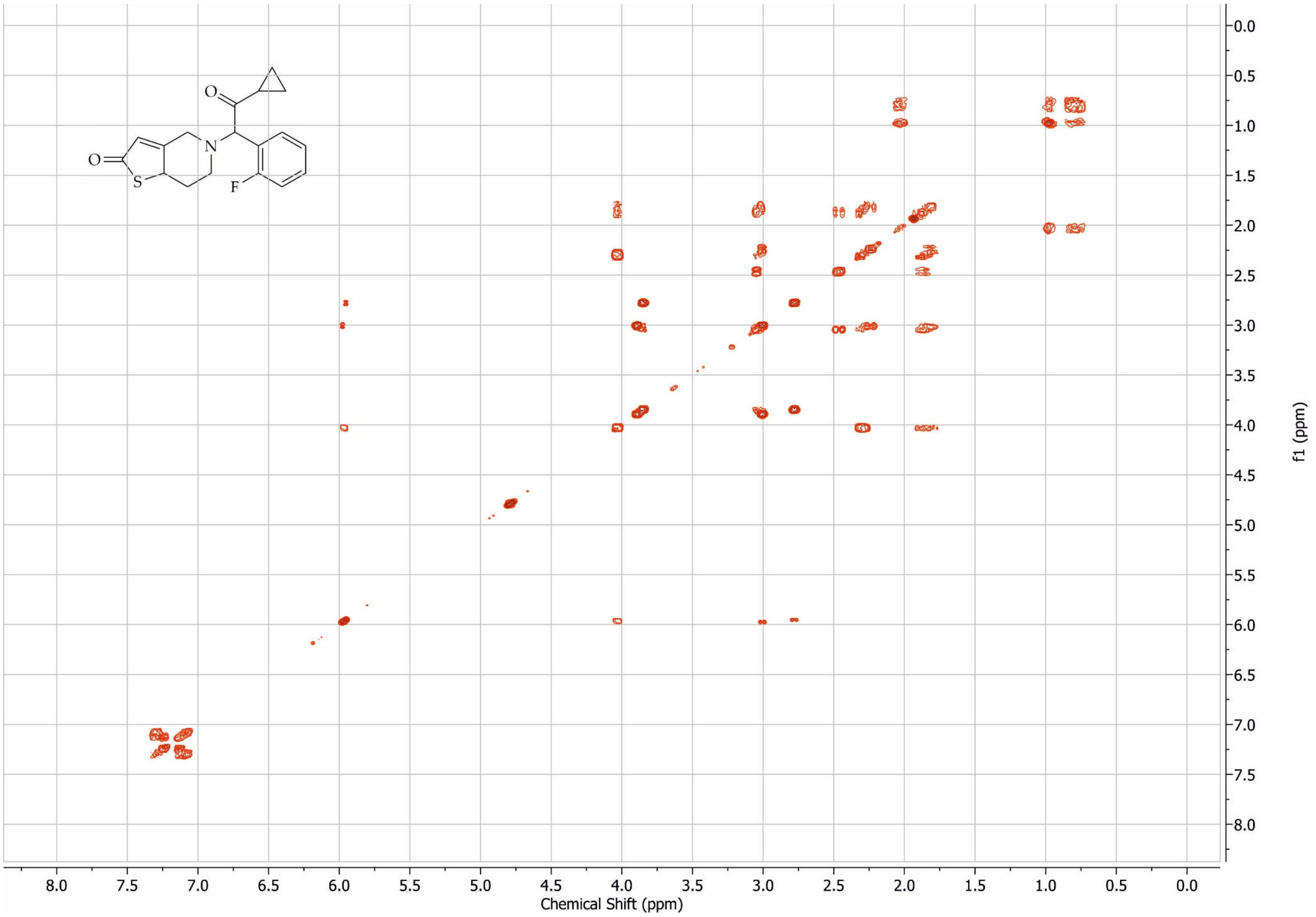

Figure S12. COSY spectrum of isolated isomers of 2-oxo-prasugrel.

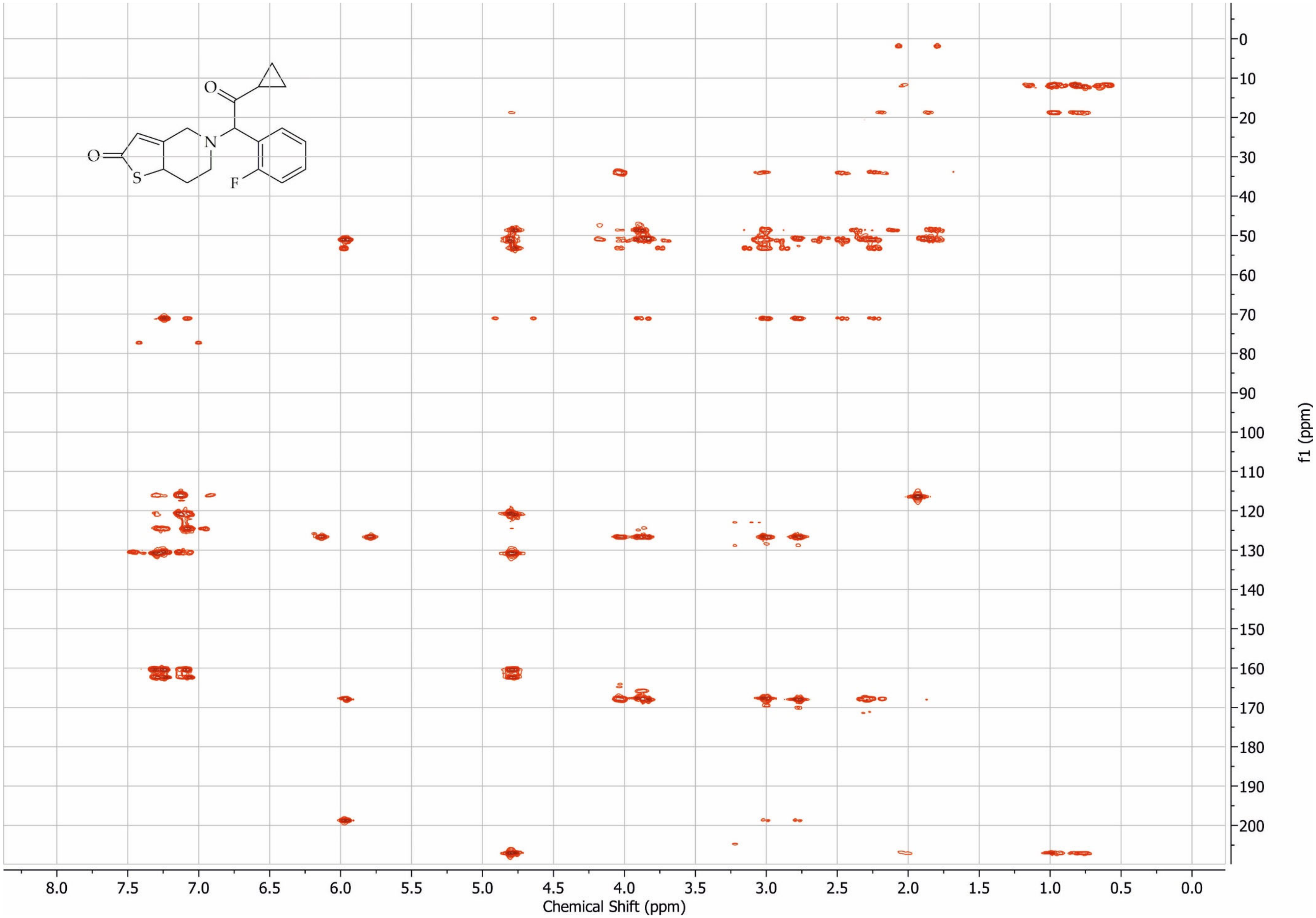

Figure S13. HMBC spectrum of isolated isomers of 2-oxo-prasugrel.

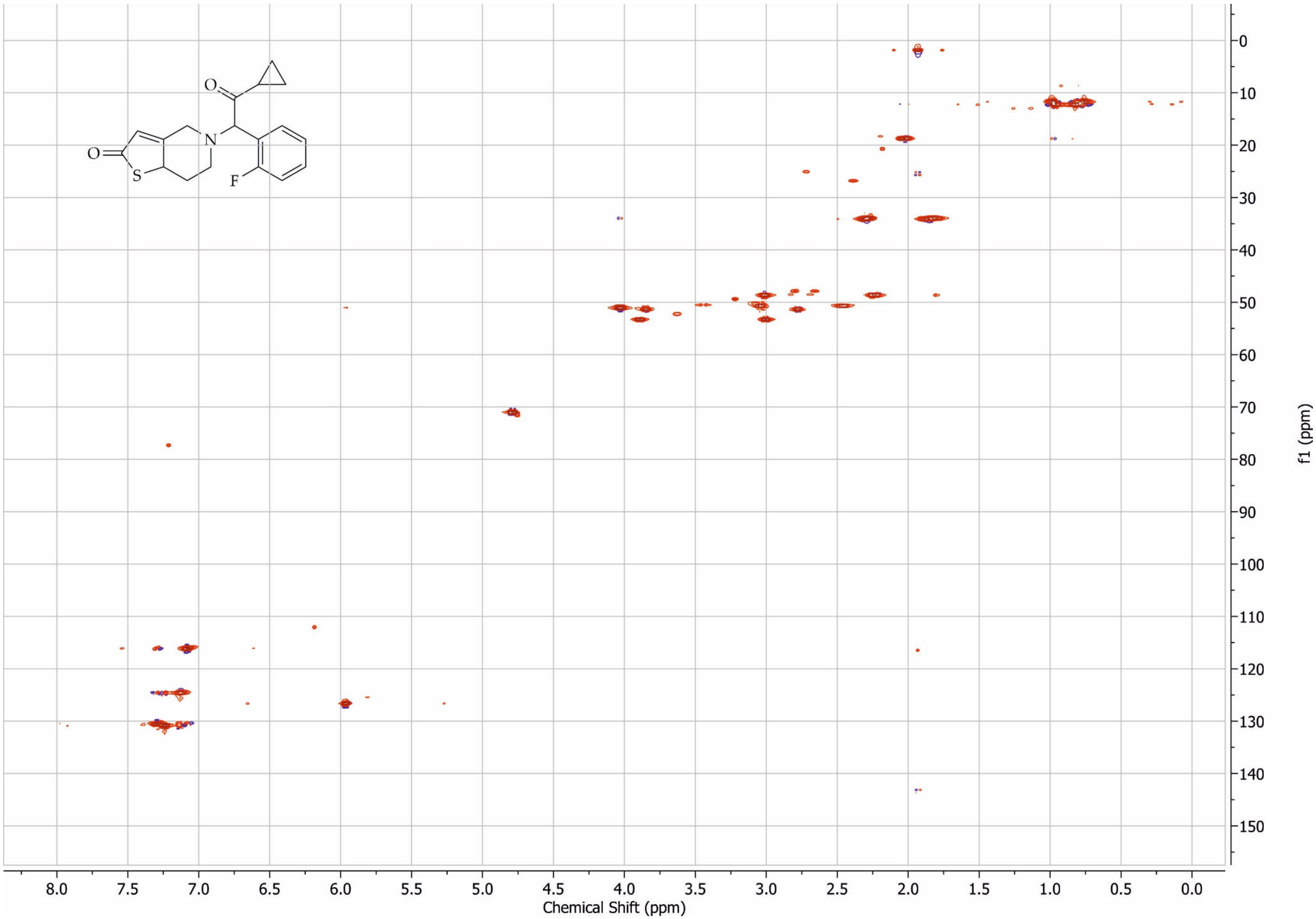

Figure S14. HSQC spectrum of isolated isomers of 2-oxo-prasugrel.

Table S2. Assignment of ^{1}H and ^{13}C NMR signals to the isomers of 2-oxo-prasugrel.

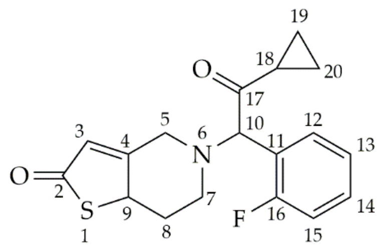

Carbon	δ ^{1}H $CDCl_3$ Isomer a	δ ^{13}C $CDCl_3$ Isomer a	δ ^{1}H $CDCl_3$ Isomer b	δ ^{13}C $CDCl_3$ Isomer b
2	-	198.80	-	198.76
3	5.98	126.70	6.00	126.64
4	-	167.97	-	167.73
5	3.88 ; 2.81	51.40	3.92 ; 3.03	53.30
7	3.04 ; 2.27	48.66	3.08 ; 2.49	50.71
8	2.34 ; 1.87	34.19	2.34 ; 1.87	33.94
9	4.06	51.07	4.06	51.08
10	4.83	71.08	4.80	71.08
11	-	120.84	-	120.84
12	7.27	130.86	7.27	130.89
13	7.16	124.62	7.16	124.62
14	7.11	116.11	7.11	116.11
15	7.33	130.51	7.33	130.51
16	-	162.33 ; 160.38	-	162.33 ; 160.38
17	-	206.96	-	207.08
18	2.06	18.76	2.06	18.81
19, 20	1.00 ; 0.82	12.29 ; 11.74	1.00 ; 0.82	12.20 ; 11.80

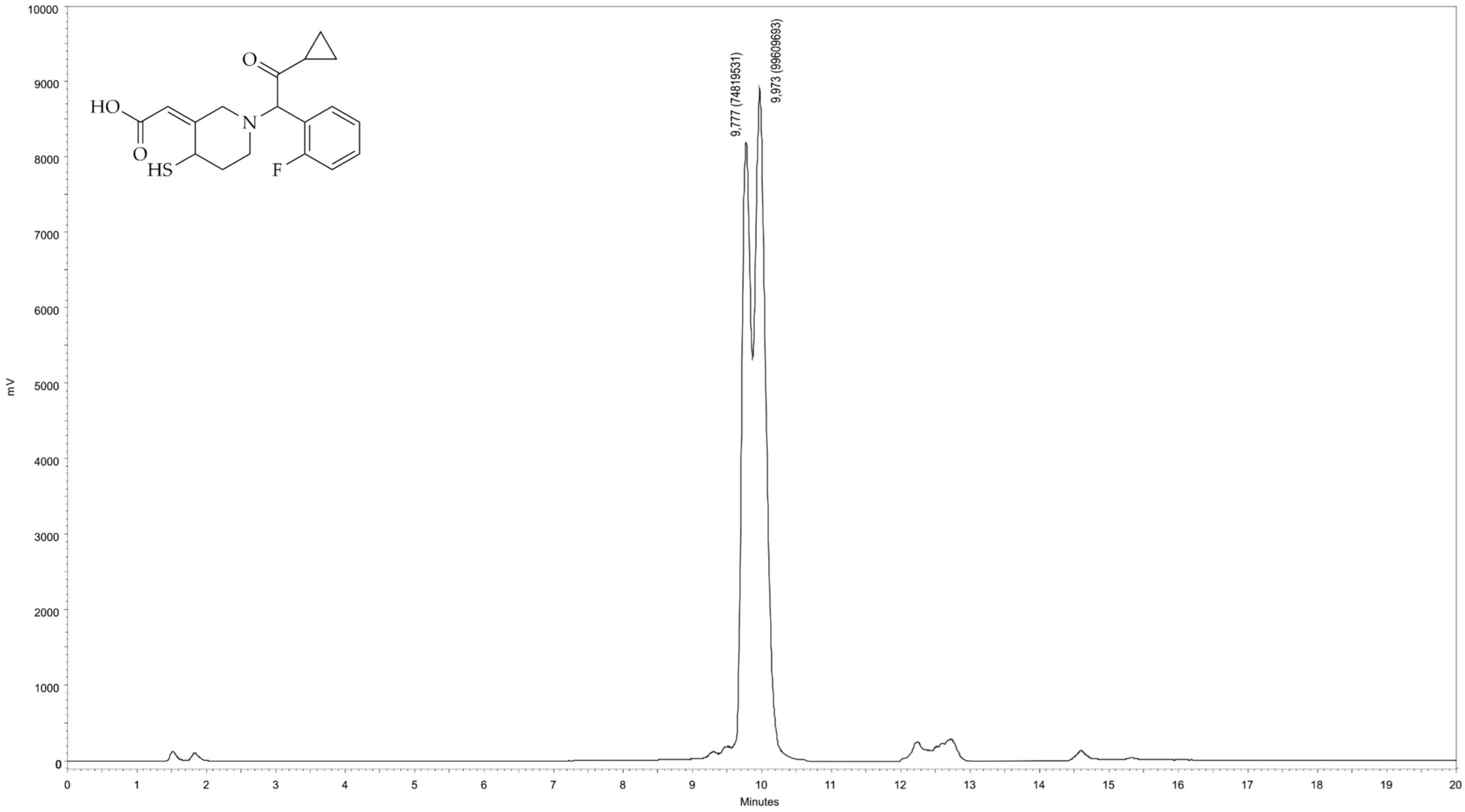

Figure S15. HPLC-ELSD chromatogram of isolated isomers of prasugrel active metabolite (PAM).

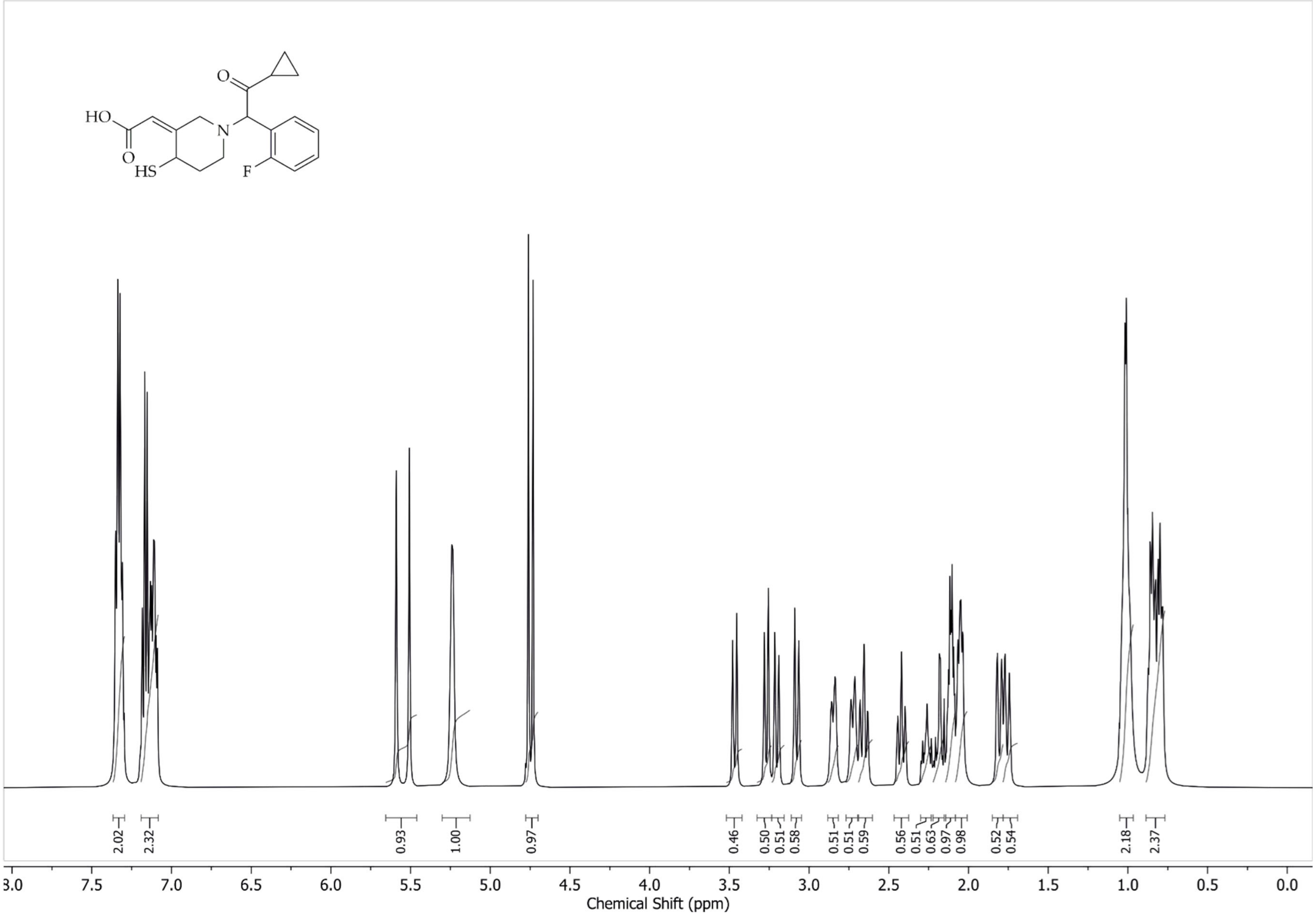

Figure S16. ^{1}H NMR spectrum of isolated isomers of prasugrel active metabolite (PAM).

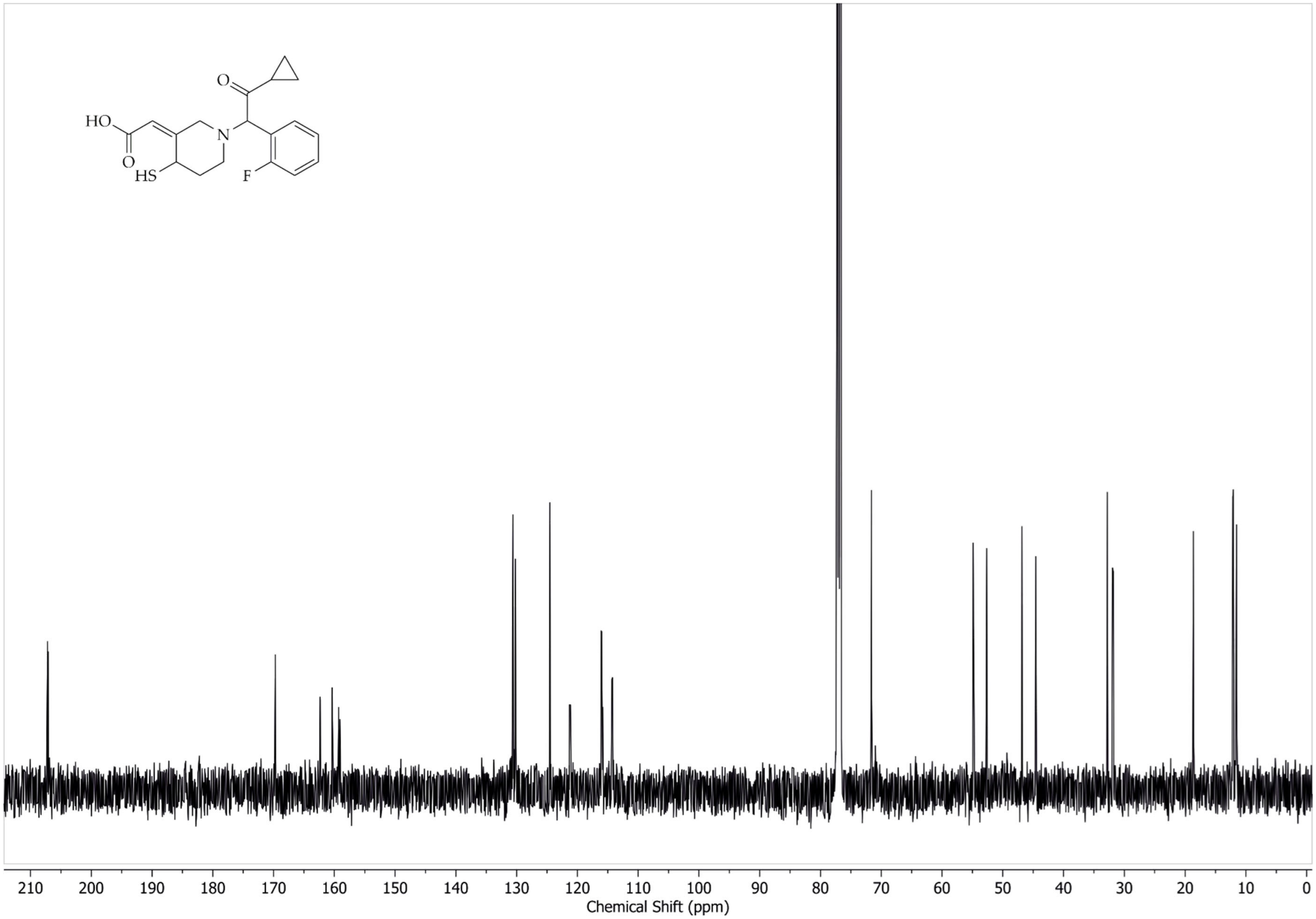

Figure S17. ^{13}C NMR spectrum of isolated isomers of prasugrel active metabolite (PAM).

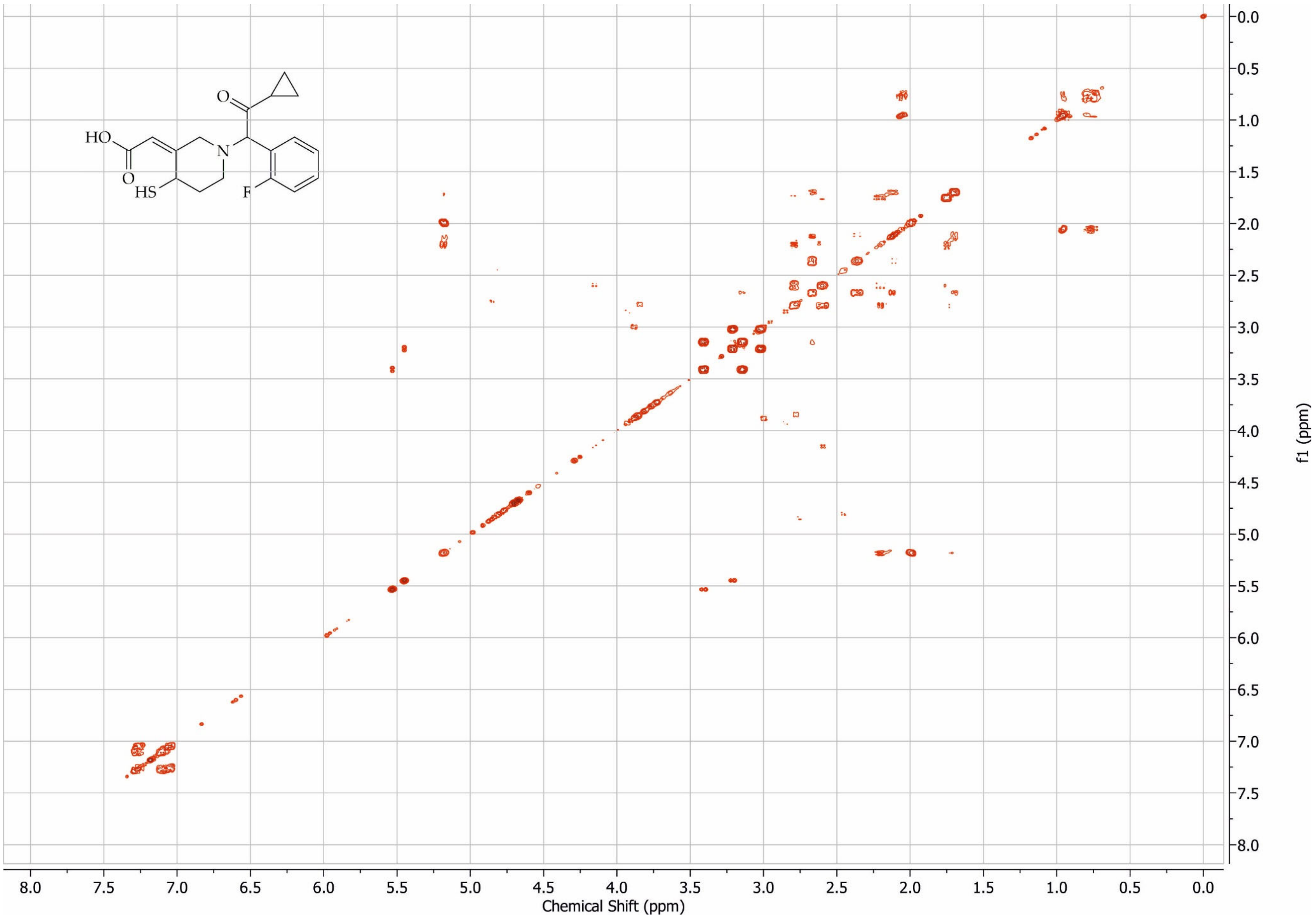

Figure S18. COSY spectrum of isolated isomers of prasugrel active metabolite (PAM).

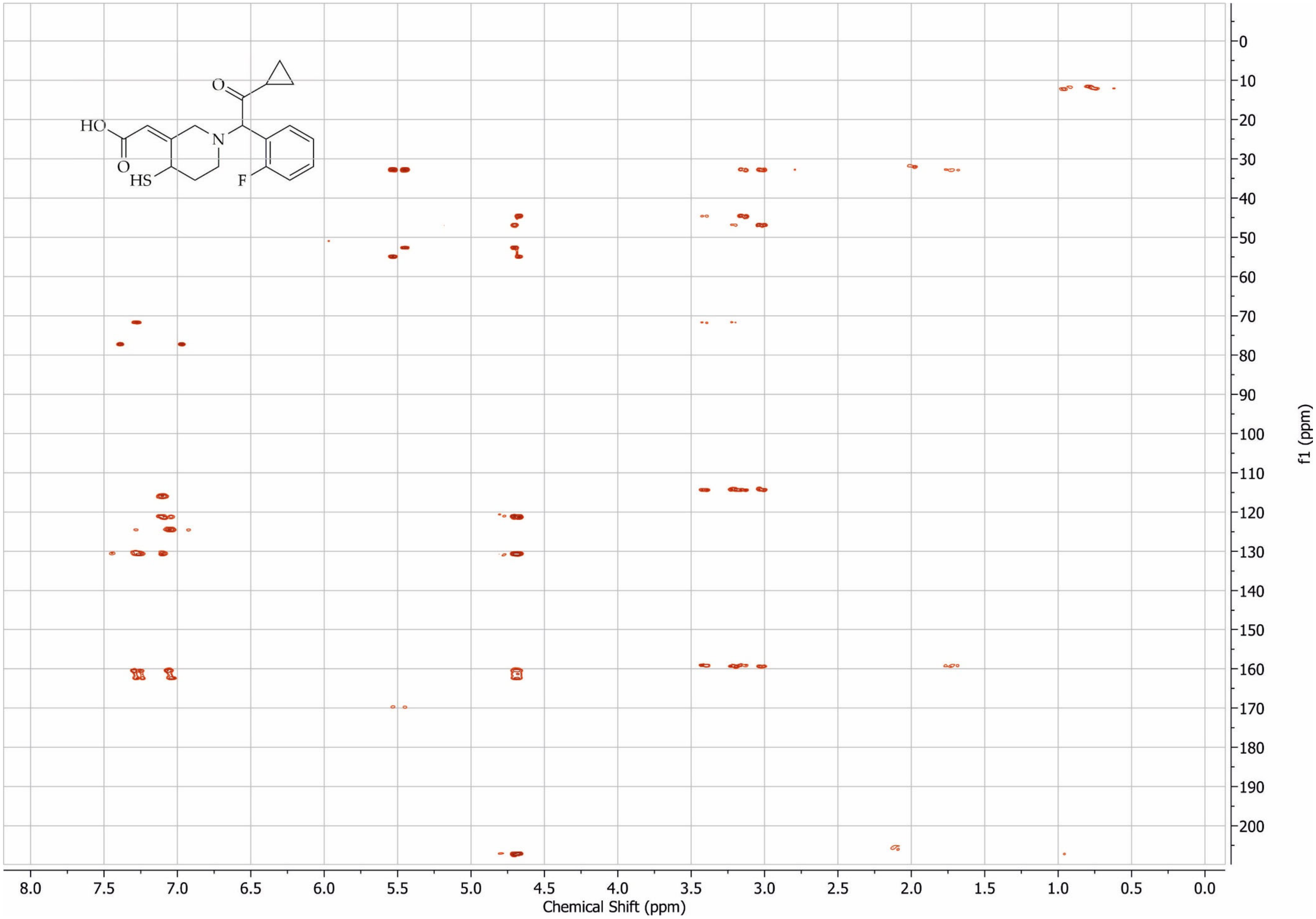

Figure S19. HMBC spectrum of isolated isomers of prasugrel active metabolite (PAM).

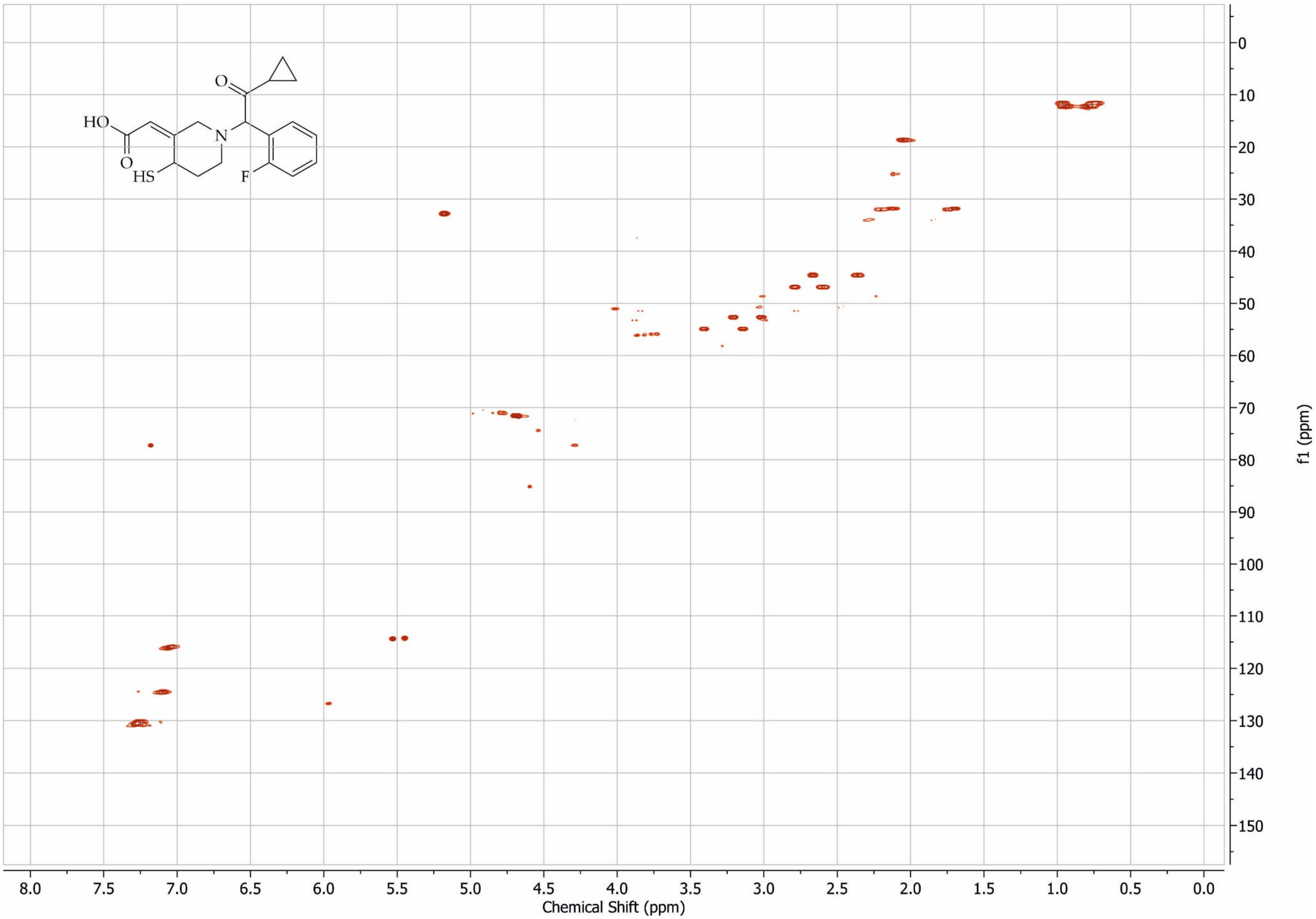

Figure S20. HSQC spectrum of isolated isomers of prasugrel active metabolite (PAM).

Table S3. Assignment of ^{1}H and ^{13}C NMR signals to the isomers of prasugrel active metabolite (PAM).

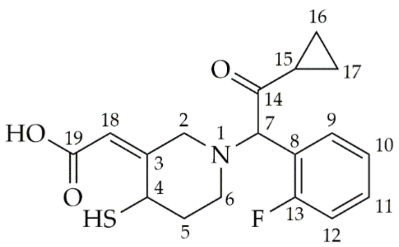

Carbon	δ ^{1}H $CDCl_3$ Isomer a	δ ^{13}C $CDCl_3$ Isomer a	δ ^{1}H $CDCl_3$ Isomer b	δ ^{13}C $CDCl_3$ Isomer b
2	3.27 ; 3.08	52.63	3.47 ; 3.20	54.87
3	-	159.30	-	159.08
4	5.24	32.82	5.24	32.82
5	2.18 ; 1.76	31.94	2.26 ; 1.81	31.81
6	2.85 ; 2.65	44.56	2.72 ; 2.42	46.83
7	4.76	71.59	4.73	71.64
8	-	121.16	-	121.16
9	7.32	130.20	7.32	130.20
10	7.17	124.54	7.17	124.54
11	7.11	115.96	7.11	115.96
12	7.34	130.62	7.34	130.62
13	-	162.33 ; 160.36	-	162.33 ; 160.36
14	-	207.21	-	207.10
15	2.11	18.61	2.11	18.61
16, 17	1.01 ; 0.82	12.11 ; 11.59	1.01 ; 0.82	12.11 ; 11.59
18	5.51	114.29	5.59	114.29
19	-	169.73	-	169.73
-SH	2.05	-	2.05	-
